# The Dual PDZ Domain from Postsynaptic Density Protein 95 Forms a Scaffold with Peptide Ligand

**DOI:** 10.1016/j.bpj.2020.06.018

**Published:** 2020-06-26

**Authors:** Nazahiyah A. Rodzli, Michael P. Lockhart-Cairns, Colin W. Levy, John Chipperfield, Louise Bird, Clair Baldock, Stephen M. Prince

**Affiliations:** 1 School of Biological Sciences, Faculty of Biology, Medicine and Health, University of Manchester, Manchester Academic Health Science Centre, Manchester, United Kingdom; 2 Wellcome Centre for Cell-Matrix Research, Division of Cell-Matrix Biology and Regenerative Medicine, Manchester Academic Health Science Centre, Manchester, United Kingdom; 3 Manchester Protein Structure Facility, Manchester Institute of Biotechnology, University of Manchester, Manchester, United Kingdom; 4 Oxford Protein Production Facility, Wellcome Trust Centre for Human Genetics, Headington, Oxford, United Kingdom

## Abstract

PSD-95 is a member of the membrane-associated guanylate kinase class of proteins that forms scaffolding interactions with partner proteins, including ion and receptor channels. PSD-95 is directly implicated in modulating the electrical responses of excitable cells. The first two PSD-95/disks large/zona occludens (PDZ) domains of PSD-95 have been shown to be the key component in the formation of channel clusters. We report crystal structures of this dual domain in both apo- and ligand-bound form: thermodynamic analysis of the ligand association and small-angle x-ray scattering of the dual domain in the absence and presence of ligands. These experiments reveal that the ligated double domain forms a three-dimensional scaffold that can be described by a space group. The concentration of the components in this study is comparable with those found in compartments of excitable cells such as the postsynaptic density and juxtaparanodes of Ranvier. These in vitro experiments inform the basis of the scaffolding function of PSD-95 and provide a detailed model for scaffold formation by the PDZ domains of PSD-95.

## Significance

Crystal structures of the first two domains (PDZ1-2) of postsynaptic density protein 95 in the apo state and ligand saturated state are shown: both crystal structures are in a compact conformation and bind glutathione. A comparison of the structures is informed by isothermal titration calorimetry measurements. Small-angle x-ray scattering reveals variations in conformation and that peptide-bound PDZ1-2 domains form a scaffold. This scaffold can accommodate segments of fellow members of the membrane-associated guanylate kinase family. The packing arrangement can propose how other proteins could integrate into the scaffold. The interpretation of the biophysical data used known crystal contacts and a space group; this approach may have general applications when similar domain types interact at high density.

## Introduction

The conduction of a signal in a nervous system requires a transmission between and within neural cells. Within a neuron, the signal is mediated by ionic conduction through the cell membrane (1), and transmission occurs between electrically responsive foci of the cell membrane. Between neurons, the signaling is mediated by intracellular junctions or synapses. A chemical synapse is formed by the close approach of two cells at a synaptic bouton. Neurotransmitter molecules (such as glutamate) released from the presynaptic membrane bind to receptors in the postsynaptic membrane. Neurotransmitter binding elicits a change in conduction through the ionotropic receptors at the postsynaptic membrane, causing an electrical signal in the form of a depolarization of the postsynaptic membrane. Electrical signals from synapses are integrated at the cell body and propagated along the neuronal axon to the terminal synapse, where neurotransmitter release is triggered. The organization of ion channels or ionotropic receptors at high densities at specialized locations in the cell membrane is fundamental to the function of neurons. These locations include the synapse, the axonal hillock, and axonal locations, such as nodes of Ranvier, in myelinated neurons.

The ionotropic receptors and ion channels at specialized locations in the neuron are coordinated and modulated by supporting protein-rich structures at the membrane surface. The postsynaptic density (PSD) is a high staining cytoplasmic layer localized at the internal surface of the postsynaptic membrane (2). The area ascribed to the PSD is of the order of 0.05 *μ*m^2^ and extends some 35–50 nm into the cytoplasm (3). An integral part of the PSD are scaffold proteins such as membrane-associated guanylate kinase (MAGUK) proteins. The disks large homolog 4 MAGUK, commonly known as SAP-90 or PSD-95, is very abundant in the PSD (4) and, in rats, has also been found at axonal juxtaparanodes (5) adjacent to nodes of Ranvier. The PSD has a laminar structure with ionotropic *N*-methyl-D-aspartate-(NMDA)-selective glutamate receptors in the membrane, which are closely associated with PSD-95 (6). Knock-down studies have shown that the PSD-95 protein plays an important role in the integrity of synapses by anchoring receptors (7). The PSD-95 protein has been studied in relation to a number of disorders of the central nervous system, including both acute and chronic conditions (8), which reflects the importance of the protein in neuronal function.

Aspects of the structural role of the PSD-95 protein in the coordination of channels and receptors have been revealed by numerous experiments; studies in mice have shown that the PSD-95 protein organizes ionotropic receptors and ion channels by forming supercomplexes on the mega-Dalton scale (9). Imaging studies have shown that PSD-95 regulates the organization of *α*-amino-3-hydroxy-5-methyl-4-isoxazolepropionic acid selective glutamate receptors in the postsynaptic membrane via the formation of domains on the nanometer scale (10). Using tomographic studies in combination with antibody labeling, separations of PSD-95 molecules in postsynaptic isolates of ∼13 nm have been observed (11). In vitro studies have shown the formation of complexes on the association with the cytoplasmic domain of an inwardly rectifying Kir2.1 potassium channel and PSD-95 (12).

**Figure 1:**
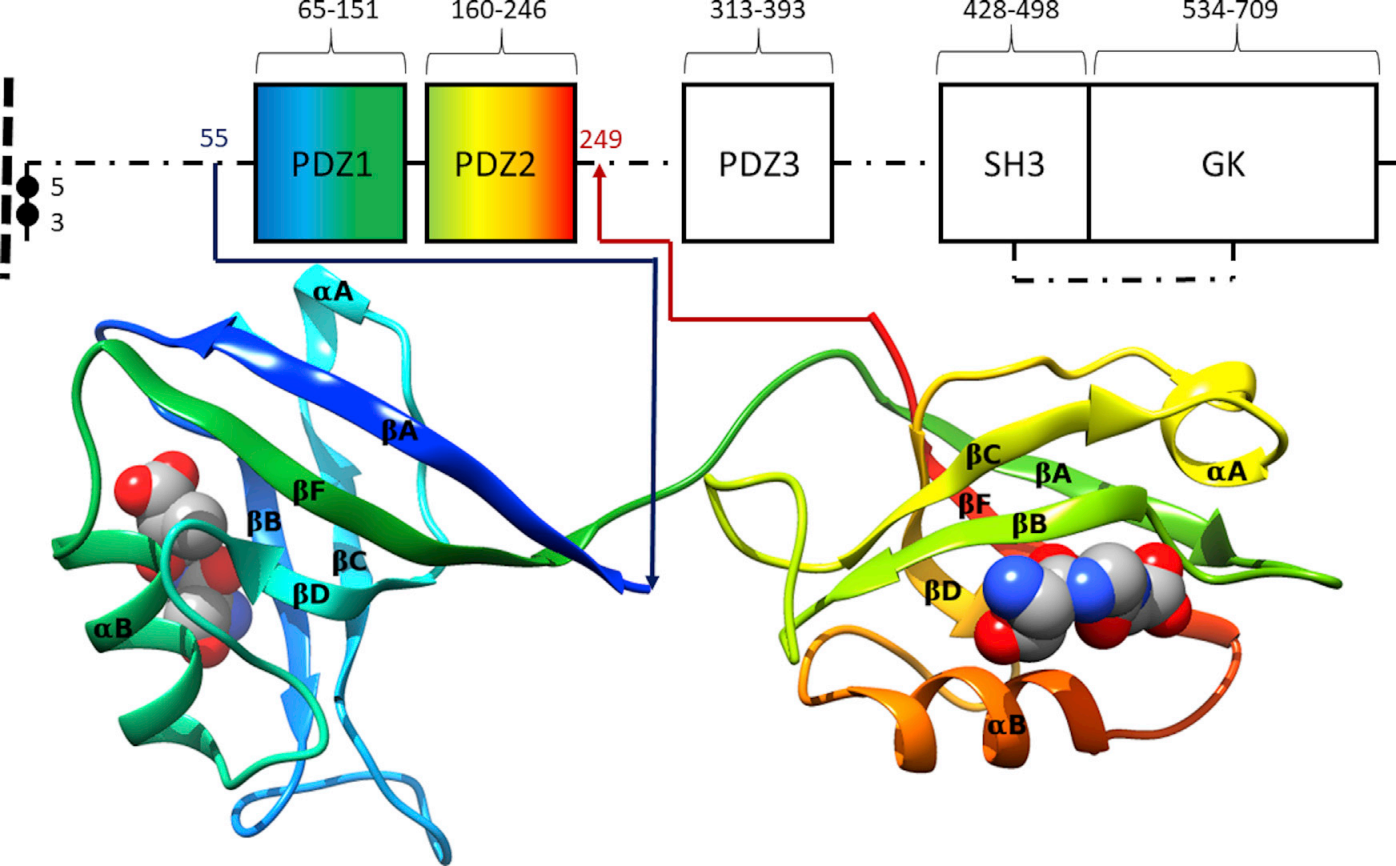
PSD-95 and the PDZ1-2 tandem domain. A schematic of the overall structure of PSD-95 is shown with PDZ1-2, SH3, and GK domains shown as boxes and interdomain linkers as dot-dash lines. The sequence numbers from the canonical sequence of human PSD-95 (UniProt: P78352) denote the domain boundaries. The dashed line indicates the membrane with palmitoylated Cys residues numbered. The PDZ1-2 domain is indicated by coloring blue to red (N- to C-terminus). An accompanying cartoon rendering of the PDZ1-2 domain, derived from the 3zrt PDB entry (25) and colored according to increasing sequence number (N-terminus, *blue*; C-terminus, *red*), shows the secondary structure of the two domains. The sequence limits of the expressed PDZ1-2 construct used in this work are shown in blue and red. Secondary structure elements are shown in a ribbon representation, and the sequence order of *β*-strands are indicated by an arrowhead. Secondary structure elements are labeled directly (16) (the nomenclature for intervening loop elements used in the text is derived with reference to these elements and is also shown explicitly in Fig. S1). A minimal type I PDZ binding ligand (Ser-Gly-Ala) is shown at each PDZ cleft in a space-fill form. The cartoon-structure diagram was produced by the UCSF Chimera program (71). To see this figure in color, go online.

PSD-95 is the best-characterized member of the MAGUK family (13,14), which also includes PSD-93, SAP-97, and SAP-102. MAGUK family proteins contain five linked domains, and both PSD-95 and PSD-93 are localized to the membrane. A schematic diagram of PSD-95 is shown in Fig. 1. Experiments in rats have shown that PSD-95 localization is via palmitoylation of Cys residues at the amino terminus (15). The canonical human isoform of PSD-95 comprises 724 amino acids (UniProt: P78352). The domains of PSD-95 are distributed within the primary sequence interspersed with linker peptides of varying lengths (Fig. 1). From the membrane-localized N-terminus, a series of three PSD-95/disks large/zona occludens (PDZ) protein domains—PDZ1, 2, and 3—are followed by closely associated SRC (kinase) homology 3 (SH3) and guanylate kinase (GK) domains.

PDZ domains consist of around 90 amino acids, and many have a characteristic Gly-Leu-Gly-Phe sequence and are sometimes named GLGF domains for this sequence. PDZ domains are abundant in the human genome and are present in over 400 proteins, often with multiple copies (16). They are also found in other organisms, having the role of presenting peptides to proteases in some (17). PDZ domains have been classified according to the properties of their cognate ligands (18,19). The PDZ domains of PSD-95 are all class I PDZ domains (18), in which the ligand is the C-terminus of the partner protein. The type I PDZ ligand has a consensus sequence of the form −X_−3_-(Ser-Thr-Cys)_−2_-X_−1_-*Φ*_0_, where X is any residue and the C-terminal residue *Φ*_0_ has an aliphatic side chain.

The way that individual PDZ domains bind their peptide ligands is well resolved because a large number of structures of PDZ domains have been determined using NMR or x-ray crystallography. The binding cleft for the peptide ligand lies between the *β*B-strand and the long *α*B-*α*-helix of each domain (Fig. 1). The GLGF signature sequence of the PDZ domain is found at the apex of the binding cleft, with GLG of GLGF in the loop between the *β*A- and *β*B-sheets (*β*A-*β*B loop) (annotated in Fig. S1). For type I PDZ domains, GLGF interacts with the C-terminus of the partner sequence (Fig. 1; (20)).

A number of ionotropic synaptic receptors and potassium ion channels possess PDZ binding motifs at their C-termini, and these have been shown to interact with MAGUKs, including PSD-95 (13). Recent studies have shown that component proteins of the synapse are able to self-assemble by separation into a protein-rich phase, and the PSD-95 protein is a component of all of these self-assembling systems (21,22). An earlier study dissected the roles of segments of PSD-95 in clustering *Shaker* Kv1.4 channels in transfected COS-7 cells (23). This work found that the N-terminal linker region is essential for clustering and that clustering is observed even for constructs truncated by the omission of C-terminal domains; for example, a construct containing only the N-terminal linker and PDZ1-2 of PSD-95 mediated clustering with wild-type efficiency. Mutation of Cys at residues 3 and 5 of an N-terminal-linker-PDZ1-2 construct was found to abolish coimmunoprecipitation of the truncated protein with full-length PSD-95. This resulted in a proposal that intermolecular disulphide linkage of residues in the N-terminal region could be a mechanism for clustering by PSD-95 (23). However, the formation of disulphide bonds in the reducing environment of the cytoplasm would require specialized enzymes and conflicts with membrane localization of PSD-95 by palmitoylation at these residues.

A number of structures of the PDZ1-2 domain of PSD-95 have been resolved before this work. A structure of the PSD-95-PDZ1-2 dual domain in complex with a peptide derived from the C-terminus of the cypin protein (sequence QVVPFSSSV) was obtained via NMR (24) (Protein Data Bank, PDB: 2ka9). The ensemble of structures deposited in this study show variation in the relative orientations and separation of domains in PDZ1-2. Two crystal structures of the PDZ1-2 fragment of PSD-95 have also been solved. A crystal structure of human apo PDZ1-2 from PSD-95 with four copies of the dual domain in the asymmetric unit is available at a resolution of 3.4 Å (25) (PDB: 3zrt). The overall conformation of all four copies of 3zrt-PDZ1-2 is essentially identical, although part of PDZ1 in one copy of 3zrt-PDZ1-2 is not resolved in the crystal structure. A crystal structure of rat PDZ1-2 from PSD-95 with two copies of the dual domain in the asymmetric unit is available at a resolution of 2.05 Å (26) (PDB: 3gsl). A ligating sequence (ETMA) derived from the C-terminus of the ionotropic glutamate receptor Glur6 is fused to the C-terminus of the 3gsl-PDZ1-2-expressed protein. This -ETMA ligand sequence is observed to associate exclusively to PDZ1 domains of 3gsl-PDZ1-2 in the crystal structure. The gross conformation of the PDZ1-2 domains in 3zrt-PDZ1-2 and 3gsl-PDZ1-2 differ by a rotation along the axis of the eight-residue interdomain linker (Fig. 1). The interdomain rotation is larger for the 3zrt-PDZ1-2 with respect to that between the two copies of PDZ1-2 in 3gsl-PDZ1-2.

Despite a detailed knowledge of the structure of the PDZ1-2 domain of PSD-95, the means by which the N-terminal segment of PSD-95 containing this dual domain can cluster channels remains unclear. The interaction of full-length PSD-95 and the tetrameric cytoplasmic domain of the inwardly rectifying potassium channel (Kir2.1) leads to the formation of extended molecular complexes, as seen using electron microscopy in negative stain (12). The aim of this study was to increase our understanding of the molecular details of this clustering by studying the essential interacting components of this complex, namely the PDZ1-2 fragment of PSD-95 and the C-terminal peptide sequence of Kir2.1, at higher resolution.

## Materials and Methods

### Protein production

All chemicals were obtained from Sigma-Aldrich (Poole, UK) unless stated.

The sequences for PSD-95 PDZ1-2 (corresponding to residues 55–249 (98–292) of UniProt: P78352 [P78352-2]), PDZ1 55–152 (98–195] and PDZ2 154–249 [197–292], and PDZ3 303–415 [346–458] were cloned into a pOPINF expression vector (27). The resulting protein is expressed with an HRV 3C protease cleavable hexahistidine tag appended at the N-terminus of the protein. Tag-cleaved proteins have Gly-Pro, followed by the protein sequence of interest. The sequence molecular weight of the PDZ1-2-cleaved construct is 20.8 kDa.

Proteins were expressed after their transformation into the *Escherichia coli* strain BL21(DE3) (New England Biolabs, Hitchin, UK). The cultures were initially propagated in double yeast-tryptone media supplemented with 50 *μ*g/mL ampicillin at 37°C in shake flasks, with 200 rpm orbital shaking. Protein expression was induced at a culture optical density at a wavelength of 600 nm of 0.6–0.8 by supplementing with 0.1 mM isopropyl-*β*-D-thiogalactopyranoside. The cultures were then incubated at 16°C for 16–20 h before cells were harvested by centrifugation at 6000 × *g*. Cell pellets were flash frozen and stored at −70°C.

The chromatography steps were carried out using an ÄKTA fast protein liquid chromatography system controlled by UNICORN 5.01 software (GE Healthcare, Amersham, UK). *E. coli* cells were resuspended in lysis buffer: 20 mM Na_2_HPO_4_-NaOH and 0.5 M NaCl (pH 8.5); for PDZ1-2, 1 mM reduced glutathione (GSH) was also included. For lysis, the buffer was supplemented with 100 mg/L DNase1 and “Complete,” Mini, EDTA-free protease inhibitor cocktail (Roche Diagnostics, Burgess Hill, UK), which was used at the manufacturer’s recommended concentration. The cell suspension was sonicated (Bandelin Sonopuls HD 3200 with a TT13/F2 probe; Bandelin Electronic, Berlin, Germany) on ice until the suspension was homogeneous. The lysate was then centrifuged at 39,000 × *g* to remove unbroken cells before the application of the supernatant to a Ni-NTA agarose resin column (QIAGEN, Manchester, UK). The affinity column was washed with a lysis buffer supplemented with 10 mM imidazole to remove weakly interacting proteins. Immobilized protein was then eluted in a stepwise manner with lysis buffer supplemented with 200 mM imidazole. Desalting chromatography was performed using a HiPrep 26/10 column (GE Healthcare) to remove the imidazole. Tag cleavage was carried out overnight at 4°C on a roller with 10 units of HRV 3C protease (Novagen; Merck Biosciences, Nottingham, UK) per milligram of protein. A negative Ni-NTA affinity purification step was carried out to remove uncleaved protein; the flow through from this step was concentrated using a centrifugal concentrator and applied to a Superdex 200 10/300 GL (GE Healthcare) size-exclusion column equilibrated with a “standard” buffer of 20 mM Tris-HCl and 150 mM NaCl (pH 8.5). PDZ1-2, PDZ1, and PDZ2 showed a single band on a Coomassie Brilliant Blue-stained (Sigma-Aldrich) SDS-PAGE gel and a single highly symmetric peak in size exclusion at a retention volume consistent with the expected molecular weight of the construct.

### X-ray crystallography

#### Crystallization and data collection

PDZ1-2 protein from a single size-exclusion chromatography (SEC) peak was pooled and concentrated with a centrifugal concentrator to a concentration of ∼0.75 mM. Vapor-diffusion, sitting-drop, 96-well crystallization plates were set up using an automated liquid handler (mosquito Crystal; TTP LabTech, Melbourn, UK). The volume ratio of the protein and screen solution was 200:200 nL. Each drop was equilibrated against a 100-*μ*L-well volume at 4°C. Crystals of unligated apo-PDZ1-2 with a maximal dimension of 250 *μ*m and a tetragonal bipyramid habit were observed after 4 weeks with a screen solution containing 0.2 M calcium acetate, 0.1 M sodium cacodylate (pH 6.5), and 40% v/v polyethylene glycol (PEG) 300.

Several attempts were made to crystallize liganded PDZ1-2 without a seeding step, but these were unsuccessful: start and ending protein concentrations in the vapor-diffusion crystallization trials were ∼0.5 and 1 mM, respectively. A large number of clear wells were seen in these crystal screens, which is consistent with the high solubility of the protein-ligand complex, limiting nucleation. PDZ1-2 crystals in the presence of ligand were obtained via matrix microseeding (28). A PDZ1-2 solution containing an excess concentration of the ligand peptide RRESEI at 98% purity (Peptide Protein Research, Funtley, UK) was prepared by supplementing a 1 mM solution of PDZ1-2 with 10 mM of the RRESEI peptide and incubating for 4 h at 4°C on a roller. Crystallization trials were set up using the mosquito liquid handling robot (TTP LabTech), with screens obtained from Molecular Dimensions (Newmarket, UK). The seed stock was prepared by mixing a drop containing apo-PDZ1-2 crystals (volume ≤ 400 nL) with 350 *μ*L of the corresponding reservoir and pulverizing the crystal using a MicroSeed Bead (Molecular Dimensions). The seed stock, protein, and crystallization screen reagent were dispensed consecutively to form drops comprising 150 nL protein solution, 50 nL seed suspension, and 200 nL reservoir, respectively. Crystals of a maximal dimension of 250 *μ*m with a tetragonal bipyramid habit were observed after 1 week in two screen solutions containing 1) 0.2M NaCl, 0.1M Na/K phosphate (pH 6.2), and 50% v/v PEG 200; and 2) 0.2M Li_2_SO_4_, 0.1M Tris (pH 8.5), and 40% v/v PEG 400.

For both apo-PDZ1-2 and RRESEI-PDZ1-2, crystals were harvested into fiber loops and flash cooled in liquid nitrogen directly from crystallization drops. Diffraction data were collected from a single crystal at the Diamond Light Source (Didcot, UK). Crystallographic data were processed using the Xia2 expert system, CCP4 software, and XDS integration software (29, 30, 31, 32, 33). Subsequent data analysis was facilitated by programs from the CCP4 suite. The crystal structure reported here for RRESEI-PDZ1-2 crystals is from the PEG 200 condition given above, which showed higher resolution diffraction (2.1 vs. 2.4 Å).

#### Crystallographic structure solution, model building, and refinement

Crystal structures of PDZ1 and PDZ2 from human SAP-97 (34) (PDB: 3rl7 and 3rl8) have been determined at high resolution in complex with a ligand peptide with sequence RHSGSYLVTSV. The two PDZ domains have a high level of sequence identity with their counterparts in PSD-95. Molecular replacement was carried out with the models prepared from one domain from each of these structures, using the Chainsaw program (35) and the sequence of the expressed construct. Molecular replacement of the apo crystal structure was effected with the program PHASER (36) and gave a solution with log likelihood gain of 771 and one copy each of PDZ1 and PDZ2 in the asymmetric unit. Clear electron density for unique residues in the individual domains was observed, as was connecting density for the linker region between the two domains. The ligand-soaked crystal structure was solved by cross-phasing using a partially refined apo-PDZ1-2 model, followed by rigid body refinement. For the crystals grown in the presence of the RRESEI ligand, a clear and unbroken electron density was seen for the main chain of ESEI residues in the initial Fo-Fc difference electron density map. Model building was carried out using the program Coot (37), interspersed with refinement using REFMAC5 (38). In the refinement process for both apo-PDZ1-2 and RRESEI-PDZ1-2, each of the PDZ domains was assigned to a rigid body, and tensors describing translation, libration, and their correlation (screw rotation) were used in REFMAC5 to describe anisotropy in the model (39). Data collection and refinement parameters are included in Table S1.

#### Isothermal titration calorimetry

Isothermal titration calorimetry (ITC) experiments were carried out using a MicroCal VP-ITC (Malvern Panalytical, Malvern, UK) instrument. All samples were prepared in the aforementioned standard buffer, and this buffer was used in control experiments to measure the heat change on dilution. A 5-mM concentration of the RRESEI ligand was placed in the syringe with PDZ1-2, PDZ1, or PDZ2 placed in the cell at a nominal concentration of 0.13 mM. ITC experiments were conducted at 25°C. After an initial priming injection, 19 consecutive 10-*μ*L aliquots were injected into the cell, each over 4.8 s, and the heat change recorded over a 250-s interval before the next injection. Data analysis was performed using OriginR 5.0 software (OriginLab; Silverdale Scientific, Bucks, UK).

### Small-angle x-ray scattering

#### Small-angle x-ray scattering data collection

Small-angle x-ray scattering (SAXS) data were collected on PDZ1-2 in two modes at two beamlines. Unfractionated SAXS data were collected on 20-*μ*L solution samples at fixed concentrations alongside the corresponding subtraction buffer. For SEC-SAXS, scattering experiments were carried out after fractionation using an Agilent 1260C HPLC system (Agilent Technologies, Stockport, UK), and data were collected on samples eluted from a 4.6-mL Shodex Kw403 silica chromatography column (Showa Denko Europe, Munich, Germany). Two collection modes were used for SEC-SAXS: 1) in which the liquid chromatography flow is paused and data images are recorded, and 2) in which the data are continuously recorded and images selected corresponding to SEC peak fractions are subsequently combined.

#### SAXS sample preparation

For apo-PDZ1-2 the protein sample was prepared in the same way as for crystallization. The buffer measured for subtraction was the standard buffer reserved from the final preparative chromatography step. In the case in which the RRESEI peptide ligand was present, the protein sample was prepared by incubation with a 10-fold excess of RRESEI in the same way described for protein crystallization. The buffer measured for RRESEI-PDZ1-2 background subtraction was also the buffer from the preparative size-exclusion step; therefore, the buffer subtraction for SAXS measurements on unfractionated RRESEI-PDZ1-2 did not remove the scattering resulting from the unbound RRESEI ligand, and a contribution due to the free ligand is present in the buffer-subtracted scattering curve. For PDZ1-2 in the presence of GSH (GSH-PDZ1-2), a sample of apo-PDZ1-2 protein at a concentration of 20 mg/mL was diluted with a 20-mM concentration of GSH prepared in standard buffer to achieve the stated GSH concentrations. GSH was also included in the subtraction buffer for these measurements.

For SEC-SAXS measurements, the protein samples were prepared as described above. The data for background buffer subtraction were selected from frames within the size-exclusion run. Here, the free ligand will be separated from a ligand-PDZ1-2 complex by the preceding in-line size-exclusion step, and therefore, any free ligand contribution to the background subtracted scattering curve is minimal.

#### Treatment of SAXS data

The scattering data were recorded on area detectors as a series of sequential images in all cases. Image data were reduced to one-dimensional scattering profiles by beamline software. These scattering profiles were compared within each data set, and those showing evidence of radiation damage (diagnosed by enhanced scattering at low q at the expense of high q scattering (40)) were excluded before the profiles were merged using PRIMUS (41). Table S2 summarizes the SAXS data sets recorded. Results are presented for unfractionated SAXS data recorded for apo-PDZ1-2, RRESEI-PDZ1-2 (June 2015 data), and GSH-PDZ1-2 (September 2016) and for SEC-SAXS with paused chromatography for apo-PDZ1-2 and RRESEI-PDZ1-2 and GSH-PDZ1-2 (January 2016). Similar results were found for the corresponding unfractionated apo-PDZ1-2 SAXS data (September 2016), and for the corresponding integrated peak SAXS data (June 2016).

#### Initial analysis of SAXS profiles

Data quality was excellent, with low noise levels even for data collected on diluted samples at higher q-values. Significant differences were observed between scattering profiles recorded for apo-PDZ1-2, RRESEI-PDZ1-2, and GSH-PDZ1-2. Initial data analysis showed that for the unfractionated SAXS data, the Guinier regions were nonlinear, particularly for RRESEI-PDZ1-2, resulting in a large error estimate for the R_g_-value determined (Table S2). The estimates of the maximal pair distance (D_max_) values obtained from the Fourier-transformed scattering data also differed according to the concentration of each sample, with larger values of D_max_ correlating with higher concentrations of PDZ1-2. The fractionated (SEC-)SAXS data indicated more well-behaved samples with smaller differences between the Guinier-derived R_g_-values (apo versus RRESEI and apo versus GSH) when compared with the unfractionated data.

#### Analysis of unfractionated apo-PDZ1-2 and RRESEI-PDZ1-2 data

To develop the best means of analysis for the SAXS data, emphasis was placed on the apo-PDZ1-2 and RRESEI-PDZ1-2 data sets collected on unfractionated samples at the Deutsches Elektronen-Synchrotron (DESY) P12 beamline (Hamburg, Germany); these data sets extend to the highest resolution (q ≤ 0.48 Å^−1^) and have the lowest noise levels (see Table S2; June 2015 data). Kratky plots of the apo-PDZ1-2 and RRESEI-PDZ1-2 SAXS data did not indicate significant overall change in the folding of the protein on addition of ligand (42). A comparison of the low q regions before and after dilution indicated that interparticle interaction was occurring in these data sets (43). There are no Cys residues in the sequence of the PDZ1-2-expressed protein; hence, the covalent association of PDZ1-2 via disulphide bonding is not possible.

A comparison of data recorded on concentrated and diluted samples of apo-PDZ1-2 revealed that Guinier regions are more linear, and D_max_-values appear lower after dilution. This indicates that interparticle affects are reduced after dilution, and in turn, the interparticle interactions are reversible. The interparticle interactions for apo-PDZ1-2 are therefore consistent with noncovalent associations between copies of PDZ1-2. The situation for RRESEI-PDZ1-2 appears more complex with a noticeably higher I(0) recorded after dilution. In contrast to this apparent anomaly in the I(0)-value, the noise level in the RRESEI-PDZ1-2 SAXS data curve is higher for the diluted data, reflecting the sample dilution made. The calculation of an absolute concentration for these data by comparison with an SAXS measurement of a bovine serum albumin standard gives values in the correct range with respect to the measured total protein concentration (see Fig. S2). However, the sample concentrations calculated from the I(0)-values for both RRESEI-PDZ1-2 and apo-PDZ1-2 reflect the apparent anomalies mentioned and are at variance with the dilutions made.

**Figure 2:**
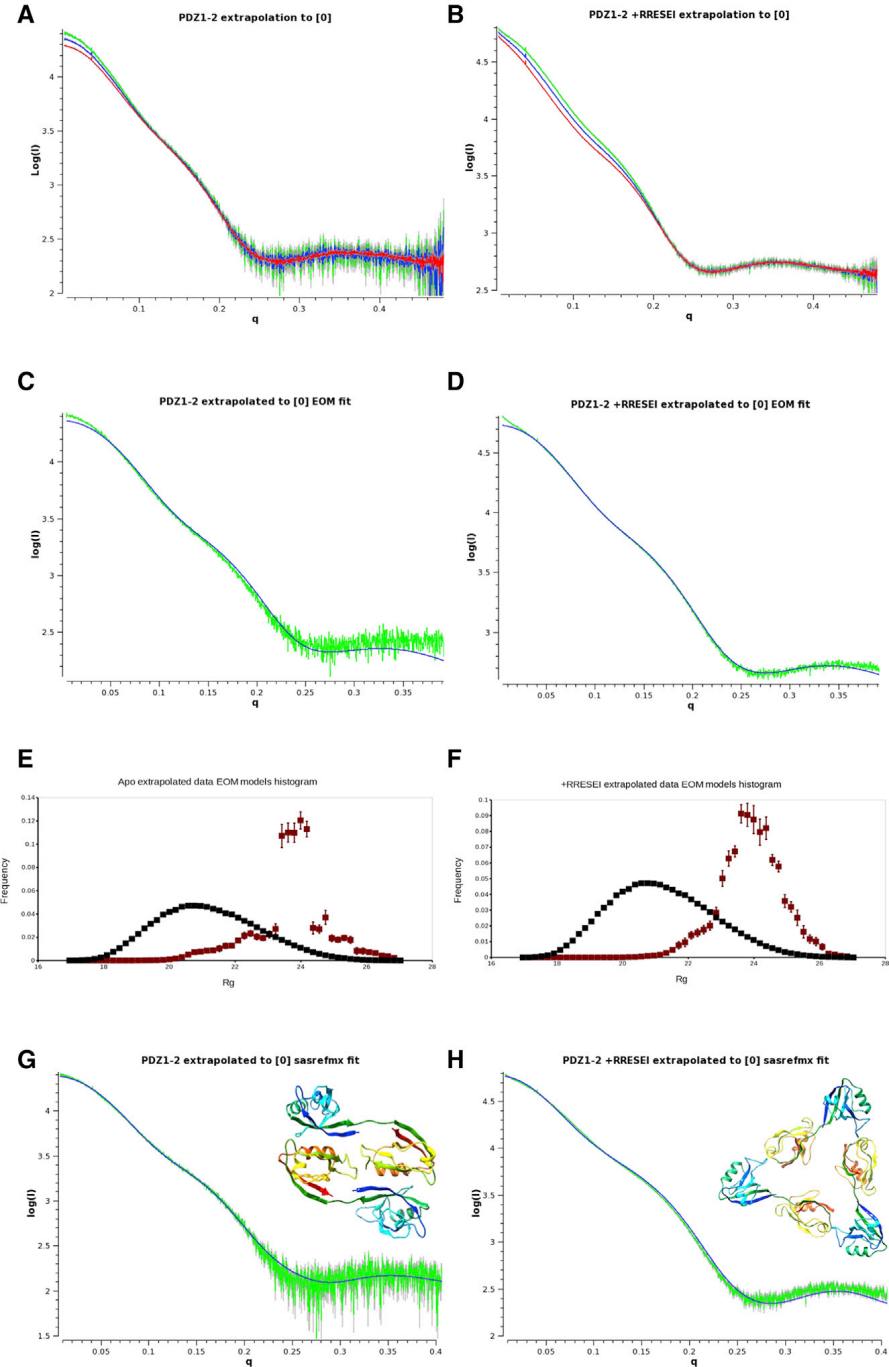
Initial model-based analysis of apo-PDZ1-2 and RRESEI-PDZ1-2 SAXS data. Scattering data are plotted as Log(I) versus momentum transfer q in Å^-1^. The extrapolation of data to infinite dilution (or zero concentration, [0]) for apo-PDZ1-2 and RRESEI-PDZ1-2 recorded at the DESY synchrotron in June 2015 (see Table S2) are shown in (*A*) and (*B*), respectively, with high concentration data shown in red, diluted data in blue, and extrapolated data in green. Before extrapolation, the high concentration and diluted data are scaled together over the q-range 0.22–0.48 Å^−1^. (*C*) and (*D*) show the ensemble model analysis fit (45) to the extrapolated data for apo-PDZ1-2 and RRESEI-PDZ1-2, respectively. The extrapolated data curve (from *A* and *B*, respectively) is in green, and calculated curves from the model ensemble are shown are in blue. The corresponding model histograms are shown in (*E*) and (*F*). The population is plotted on the ordinate and R_g_ on the abscissa; the black points are pool models, and red points are models selected from the pool; error bars are calculated as the standard deviation of 20 duplicate runs. (*G*) and (*H*) show monomer and oligomer refinements for apo-PDZ1-2 and RRESEI-PDZ1-2, respectively (46). Extrapolated data (from *A* and *B*) are shown in green, fitted curve in blue; in each case, an extended monomer (3zrt-PDZ1-2) is refined alongside the oligomer shown as an inset. To see this figure in color, go online.

The effects of interparticle interference on SAXS measurements can be alleviated by a projection to infinite dilution (43). This empirical calculation proceeds via the scaling of dilutions at higher values of q in which the effects of interparticle interference are minimal, followed by a point-by-point linear projection of I(q)-values. In this calculation, the dilution factor can be used rather than the absolute concentrations. This process is shown in Fig. 2, *A* and *B* for apo-PDZ1-2 and RRESEI-PDZ1-2, respectively. The scaled scattering curves for both RRESEI-PDZ1-2 and apo-PDZ1-2 show an increase of intensity at lower q-values after dilution (Fig. 2, *A* and *B*); this effect is evident over a considerably larger q-range for RRESEI-PDZ1-2 compared with apo-PDZ1-2. The projected SAXS curves showed more linear Guinier plots and more precision in the value of R_g_ for RRESEI-PDZ1-2 (Fig. S3).

Initial analysis was therefore performed on apo-PDZ1-2 and RRESEI-PDZ1-2 extrapolated to zero concentration to minimize any interparticle effects. However, it should be noted that the P(r) distributions calculated from the extrapolated apo-PDZ1-2 and RRESEI-PDZ1-2 data still showed some evidence of interparticle interference, with P(0) > P(D_max_) (Fig. S4).

#### Ab initio analysis of apo-PDZ1-2 and RRESEI-PDZ1-2 scattering curves projected to zero concentration

Dummy atom models of apo-PDZ1-2 and RRESEI-PDZ1-2 were constructed using the program DAMMIF (44). The agreement of the dummy atom models with the data curves was reasonable, although data were truncated (q ≤ observed R_g_/8) by DAMMIF to allow modeling. For data collected in the presence of RRESEI, the data range was 0.015 < q < 0.300 Å^−1^, and the model agreement Χ^2^-value was 8.7; in the apo case, the range was 0.011 < q < 0.204 Å^−1^, Χ^2^ = 6.7. The dummy atom models each gave a dumbbell-like-shaped envelope that would accommodate a PDZ1-2 model. The envelope was more compact in the case of apo-PDZ1-2 compared with RRESEI-PDZ1-2, and both have an additional projection that could not be accounted for with a single PDZ1-2 model (Fig. S5).

#### Model-based analysis of apo-PDZ1-2 and RRESEI-PDZ1-2 scattering curves projected to zero concentration

The dummy atom envelopes obtained for apo-PDZ1-2 and RRESEI-PDZ1-2 data extrapolated to zero indicated that different or multiple conformations of the dual domain may be present; therefore, the model-based analysis using ensemble optimization method (EOM) (45) software was undertaken. The analysis used the same extrapolated data with a limit of q ≤ 0.4 Å^−1^. The EOM analysis gave different histogram distributions of models for apo-PDZ1-2 and RRESEI-PDZ1-2; a sharper R_g_ histogram distribution was observed for apo-PDZ1-2 data compared with a broader population for RRESEI-PDZ1-2 (Fig. 2, *E* and *F*); the corresponding histograms for D_max_ show similar trends (Fig. S6). The ensemble analysis gave a clear indication that there were different domain separations present in the SAXS data. The presence of two or more particular conformations of PDZ1-2 was seen in the derived models. The agreement of the EOM analysis was best over the mid-q-range of the curve and poorer at higher and lower q-values (Fig. 2, *C* and *D*). The representative models generated by EOM generally contained a model with R_g_ of around 25Å with a high weight assigned alongside one or more compact models (R_g_ in the range 21–24 Å).

An alternative explanation for the distributions seen in the ensemble analysis is the formation of intermolecular complexes. A very compact structure (R_g_ ∼21.2 Å) consistently assigned in the ensemble analysis of apo-PDZ1-2 is not represented by the crystal structure reported here or any of the preceding crystal structures (25,26). This domain configuration could arise from the close approach of two PDZ domains from separate PDZ1-2 molecules occurring on the formation of oligomers. Should these intermolecular interactions be spatially specific in nature, their effects would not be removed by projection of SAXS curves to infinite dilution.

#### Construction of oligomer models for SAXS fitting

Oligomer models were constructed from combinations of a known conformation of PDZ1-2 and known intermolecular interfaces present as contacts in x-ray crystal structures. All of the PDZ1 and PDZ2 domains in both the 3gsl and 3zrt crystal structures take part in the same PDZ1-PDZ2 heterodimeric interaction (shown in Fig. S7 *A*). The *α*B-helix adjacent to the unoccupied peptide binding cleft of each PDZ2 domain associates with the *β*D-*β*E loop from PDZ1. The significance of this interaction is shown by the fact that the 3zrt structure can be re-solved by molecular replacement using an ensemble made from the two PDZ1-PDZ2 heterodimers present in the asymmetric unit of 3gsl; using the CCP4 program PHASER (36) and the deposited crystallographic data, a log likelihood gain of 1337 was obtained for four copies of the ensemble; the log likelihood gain value may be enhanced by the noncrystallographic symmetry implied by the presence of multiple copies of the heterodimer, but it is much greater than the threshold of eight, which indicates a correct solution. Therefore, this *α*B(PDZ2)-*β*D-*β*E(PDZ1) interface was used as an oligomeric contact. One obvious oligomer is the double dimer, of which there are two copies in the asymmetric unit of 3zrt-PDZ1-2.

An intermolecular contact was found in the 3gsl structure with the *α*A-helix of PDZ2 forming a contact with the *β*B-*β*C loop of PDZ1 (in 3gsl, a C*α*-C*α* distance of 5.3 Å is obtained between Ala 199 in chain A and Asp 90 of chain B after the application of the symmetry operation 1 − x, 1/2 + y, and −z). This *α*A(PDZ2)-*β*B-*β*C(PDZ1) crystal contact (shown in Fig. S7 *B*) was also used as an oligomeric contact. On combining the *α*A(PDZ2)-*β*B-*β*C(PDZ1) interaction with the 3zrt-like conformation of PDZ1-2, it was clear that a trimeric complex could also form.

Initial modeling of apo-PDZ1-2 and RRESEI-PDZ1-2 SAXS curves projected to zero concentration was undertaken using these models. Monomer and oligomer fractions were refined using the ATSAS program SASREFMX (46), and encouraging results were obtained for both the apo-PDZ1-2 and RRESEI-PDZ1-2 curves projected to zero concentration (Fig. 2, *G* and *H*). In the apo-PDZ1-2 case, the combination of 3zrt-PDZ1-2 along with a dimer of the same (similar to one half of the asymmetric unit of the 3zrt-PDZ1-2 crystal structure) (Fig. 2 *G*, *inset*) was effective in SAXS curve fitting at low-medium resolution (Fig. 2 *G*, *main plot*) and gave Χ^2^-values of ∼16. In the case of RRESEI-PDZ1-2, the 3zrt-PDZ1-2 conformation plus a trimer of the same (Fig. 2 *H*, *inset*) was similarly effective (Χ^2^-values of ∼28; Fig. 2 *H*, *main plot*). The SASREFMX analysis also provided a refined model for a trimer of RRESEI-PDZ1-2.

#### Model-based analysis of apo-PDZ1-2 and RRESEI-PDZ1-2 recorded at fixed concentrations

EOM analysis of the SAXS data recorded at fixed concentrations (∼0.72 and 0.36 mM) for apo-PDZ1-2 gave good agreement over the midrange of data (Χ^2^-values for the concentrated data ∼5). However, the EOM histograms gave bifurcated distributions of models with different weights of the two peaks for different concentrations. The minor peak at R_g_ 22.5 Å shown for the apo extrapolated data histogram (Fig. 2 *E*) is increased at the expense of the major peak around 24 Å. In the case of the RRESEI-PDZ1-2 curves recorded at fixed concentrations (∼0.72 and 0.36 mM), the agreement obtained with the data by EOM was very poor (Χ^2^-values for the 0.72 mM data > 999), with large differences at both low and high q. The model histograms gave a single broad peak at each concentration, with the histogram peak skewed toward higher R_g_-values for the data at a higher concentration.

The application of the simple monomer or trimer oligomer model was more effective in fitting the RRESEI-PDZ1-2 curves at fixed concentrations, giving a marked improvement in agreement (Χ^2^-values ∼60 and 30 for 0.72 and 0.36 mM scattering curves, respectively) over the EOM-based analysis. Additionally, the oligomer models could be extended by combining the interfaces used in the fitting of the extrapolated curves. These combinations used the dimer interface (Fig. 2 *G*, *inset*) along with the trimer determined by SAXREFMX (Fig. 2 *H*, *inset*) to generate extended oligomers. A trimeric oligomer of extended PDZ1-2 can interact with a copy of itself through the formation of a dimer of trimers, with a local interface equivalent to the double dimer (Fig. 2 *G*, *inset*). These interactions could then be propagated to form more extended oligomers, including a trimer of trimers with threefold screw-rotational symmetry. Further combinations of these trimers indicated that extended linear and branched arrays can form. The inclusion of these types of oligomers in the SAXS fitting of RRESEI-PDZ1-2 data at fixed concentrations using the ATSAS program OLIGOMER (41,47) again improved the fitting of the data.

Overall, it was found that the fitting of SAXS curves for apo-PDZ1-2 and RRESEI-PDZ1-2 using either an ensemble- or oligomer-based approach alone is unsatisfactory. However, the two techniques are complementary; the inclusion of oligomers improves the fit at a low q, and interdomain contacts such as *α*A(PDZ2)-*β*B-*β*C(PDZ1) can account for the very compact PDZ-PDZ interaction (R_g_ = 22.1Å) seen in the EOM analysis, whereas the fit of the midrange of each curve is improved by including variation within the dual domain structure of PDZ1-2 derived from ensemble analysis.

#### Assignment of an I2_1_3 space group to the packing arrangement of the PDZ1-2 oligomers

To enable a systematic approach of the use of oligomers in SAXS data fitting, a space group was assigned to the packing of PDZ1-2 oligomers. Exploring the structures formed by the association of trimers of PDZ1-2 described above revealed the presence of parallel threefold rotation and 3_1_ screw-rotation symmetry axes and, separately, parallel twofold rotation axes each in spatially distinct directions. These symmetry elements are present in multiple directions only in cubic space groups (numbers 195–230 in *International Tables for Crystallography: Volume A* (48)). From the set of 13 chiral cubic space groups, those possessing 4 or 4_1_ axes could be eliminated. Because the threefold axes do not intersect in the extended oligomers and twofold axes are present, the only possible space group conforming to the symmetry elements encountered is I2_1_3 (number 199) (48).

The unique unit cell parameter (a) for the cubic lattice and the position of the PDZ1-2 domain with respect to the coordinate origin then required definition. This was accomplished via generating a trimeric arrangement of 3 PDZ1-2 double domains. Initially, a model of the PDZ1-2 double dimer was obtained; PDZ1 and PDZ2 domains from the RRESEI-PDZ1-2 crystal structure were docked onto the 3zrt-PDZ1-2 conformation, and restrained refinement of this structure against RRESEI-PDZ1-2 SEC-SAXS data was carried out using SASREFMX. Interdomain restraints were obtained from the *α*B(PDZ2)-*β*D-*β*E(PDZ1) interface in the 3gls-PDZ1-2 crystal structure, and a monomer and symmetric dimer model was refined against the SAXS data. The resulting refined dimer was similar in form to those found in the 3zrt crystal structure but with a slightly increased twist of the dimer about the interface perpendicular to the dimeric twofold rotational axis. The twofold axis of this dimer was then oriented with reference to a set of mutually perpendicular right-handed axes, *x*, *y*, and *z*, such that the dimer twofold axis was in a direction perpendicular to the *z*-*y* plane and intersected the *x* axis. A trimer of dimers can then be generated by rotation about the resultant vector of the *x*, *y*, and *z* axes. The rotation of the dimer about the twofold axis and the displacement of the dimer from the *z*-*y* plane *Δ*x were adjusted so that the contact between dimers in the trimer was similar to the *α*A(PDZ2)-*β*B-*β*C(PDZ1) crystal contact found in 3gsl-PDZ1-2 (Fig. S7 *B*). The position of PDZ1-2 was then fixed as any single copy of PDZ1-2 within the resulting trimer of dimers. The unit cell length |**a**| could then be defined as 4 × (*Δ*x) = 148 Å. The precision of this process is dependent upon the accuracy of the dimer model and the positioning of this model in space. When generating the optimal trimer of dimers model, the interval of rotation about the twofold axis was 5°, and the interval in *Δ*x was 1 Å. The imprecision is therefore likely to be dominated by the error in the SASREFMX refinement of the dimer, which was limited to a q(max) of 0.4 Å^−1^—on this basis, the error is estimated to be (2*π*)/(2 × q(max)) ≈ 8 Å.

Any packing arrangement compatible with the I2_1_3 space group can be faithfully reproduced based on a single copy of the extended PDZ1-2 molecule. The two intermolecular contacts, namely the *α*B(PDZ2)-*β*D-*β*E(PDZ1) and the *α*A(PDZ2)-*β*B-*β*C(PDZ1) contacts (Fig. S7, *A* and *B*), are features of the packing. For the final model, the *α*B(PDZ2)-*β*D-*β*E(PDZ1) PDZ1-PDZ2 dimer was redocked into the unit cell to preserve the fidelity of the *α*B(PDZ2)-*β*D-*β*E(PDZ1) interface precisely. Minor side-chain clashes in models were resolved by adjusting side-chain rotamers in Coot, followed by geometrical refinement in REFMAC5.

## Results

### X-ray crystallography

**Figure 3:**
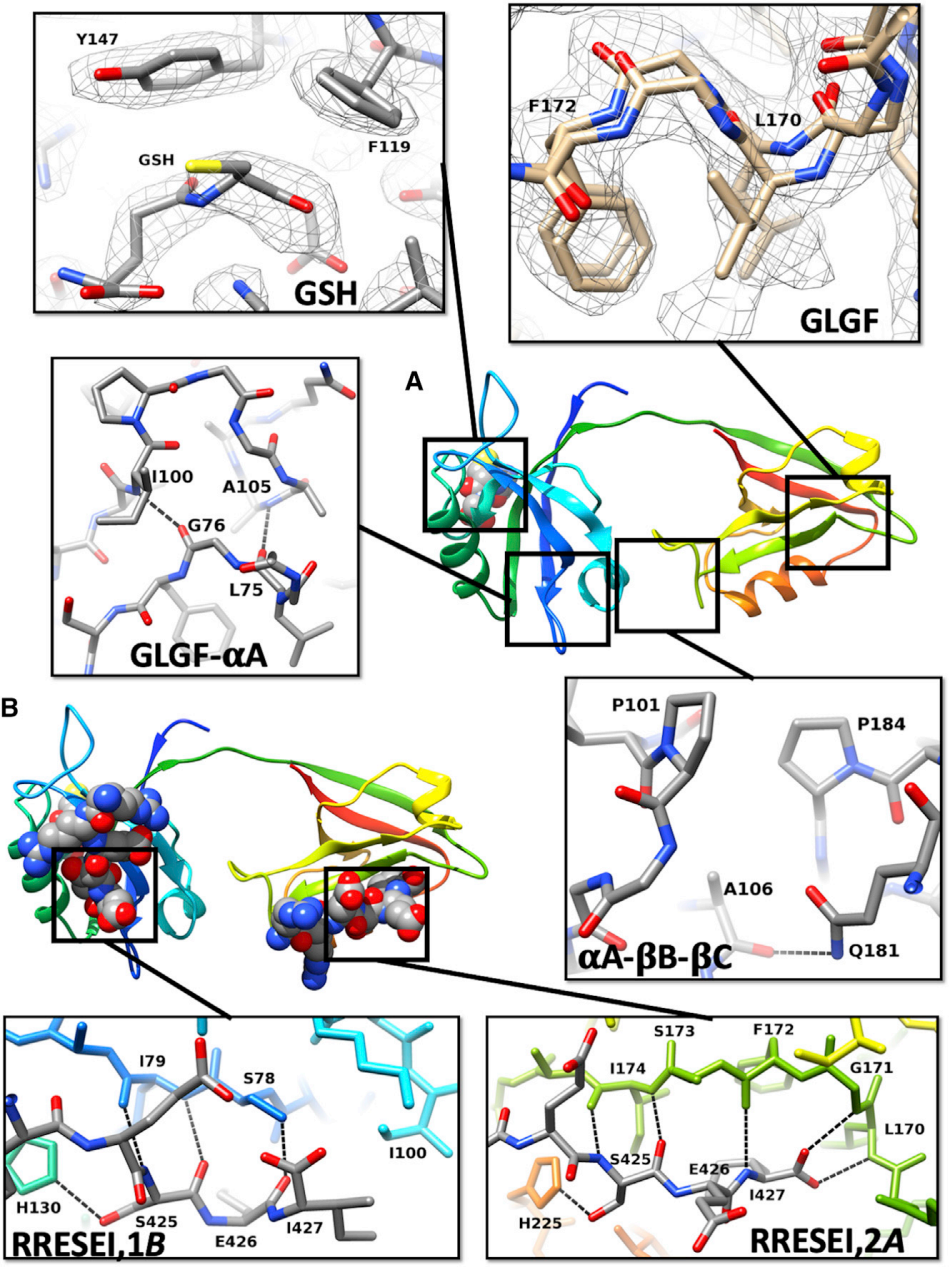
Crystal structure of PDZ1-2. The crystal structures of apo-PDZ1-2 and RRESEI-PDZ1-2 are shown in the same orientations in (*A*) and (*B*), respectively; the PDZ1-2 double domains are represented in a similar way to Fig. 1, with the RRESEI (in *A*) and GSH (in both *A* and *B*) ligands shown in a space-filled form. The protein is colored in a rainbow fashion from the N-terminus to C-terminus with PDZ1 in blue and green and PDZ2 in green and red. Callout panels from (*A*) indicate detailed elements of specific regions of the crystal structures of apo-PDZ1-2 with labeled residues and PDZ1-2 covalent bonds colored according to atom species (C, *gray*; N, *blue*; O, *red*). The panel labeled “GSH” shows the chicken-wire 2Fo-Fc density assigned to GSH from apo-PDZ1-2 (0.2 e^−^Å^−3^); “GLGF” shows the dual conformation of the GLGF motif of PDZ2 alongside 2Fo-Fc electron density (contour at 0.1 e^−^Å^−3^) (the dual conformation of the whole *β*A-*β*B loop is shown in Fig. S8*B*); “GLGF-*α*A,” shows the hydrogen bonds between the GLGF motif and residues in the *α*A-helix of PDZ1; and “*α*A-*β*B-*β*C,” shows the intramolecular contact between PDZ1 and PDZ2 (also shown with a surface in Fig. S8*C* to further demonstrate the water excluding interaction between Pro 101 and Pro 184). Callout panels from (*B*) indicate detailed elements of specific regions of the crystal structure of RRESEI-PDZ1-2 with labeled residues; PDZ1-2 is colored in the same way as the RRESEI-PDZ1-2 cartoon in (*B*), and the ligand residue covalent bonds are colored according to atom species (C, *gray*; N, *blue*; O, *red*). The panel labeled “RRESEI,2*A*” shows one conformation of RRESEI binding to PDZ2; here, the terminal Ile of RRESEI is inserted into of the cleft in the vicinity of F172. Panel “RRESEI,1*B*” shows the alternate conformation of RRESEI associated with PDZ1; here, the terminal Ile lies outside of the cleft in the vicinity of I100. Hydrogen bonds are indicated by dotted lines in all panels. To see this figure in color, go online.

The interaction of a peptide with both PDZ domains of PDZ1-2 is only resolved in detail using a high-resolution structural technique; hence, both PDZ1-2 alone and PDZ1-2 plus a ligand peptide were studied using x-ray crystallography. A sequence of RRESEI corresponding to the last six residues (422–427) of Kir2.1 was chosen. The ESEI sequence comprises a type I PDZ domain interaction motif, and the preceding RR residues were included to ensure that a peptide amino terminus does not interfere with ligand association at the PDZ cleft. These RR residues have also been implicated in receptor trafficking (49). The RR residues ensure overall charge neutrality and may enhance peptide solubility. Crystal structures of apo-PDZ1-2 and RRESEI-PDZ1-2 state were obtained at resolutions of 2.0 Å (R_free_ 26.0%) and 2.1 Å (R_free_ 23.8%), respectively (see Table S1). The crystal structures obtained are in the same tetragonal space group with a very similar unit cell. The same gross PDZ1-2 double-domain conformation is present in both apo-PDZ1-2 and RRESEI-PDZ1-2 (Fig. 3, *A* and *B*). The PDZ1-2 complex has a direct intramolecular contact between PDZ1 and PDZ2 formed by interactions between the *β*B-*β*C loop of PDZ2 and the *α*A-helix of PDZ1 (the loop annotation is shown in Fig. S1). A hydrogen bond is formed between the main-chain amide O of Ala 106 and the side-chain amide NH_2_ of Gln 181 (N*ε*2-O distance of 3.0Å) (Fig. 3 *A*, *αA-βB-βC*), and there is a direct interaction between Pro 101 and Pro 184 (Fig. 3 *A*, *αA-βB-βC*; Fig. S8 *C*). The *α*A(PDZ1)-*β*B-*β*C(PDZ2) interaction results in a more compact conformation of PDZ1-2 in the structures reported here compared with those found in 3zrt-PDZ1-2 and 3gsl-PDZ1-2 (Fig. S9).

For RRESEI-PDZ1-2, the binding cleft of both PDZ domains is ordered, and each binding site shows electron density for the RRESEI peptide. All of the ligand residues could be fitted, but the two Arg residues had markedly weaker electron density, indicating that alternate conformations of these residues could be present. Similar interactions between the bound peptide and the clefts of both PDZ1 and PDZ2 are seen. The main-chain amide linkages of the Ser 425 residue of the incoming RRESEI peptide make H-bonds with the *β*B-strand of both PDZ domains (Fig. 3, *RRESEI,1B*; Fig. 3, *RRESEI,2A*). A hydrogen bond is formed between the Ser alcohol and a His side chain (PDZ1-130 and PDZ2-225) on the *α*B-helix (Fig. 3, *RRESEI,1B*; Fig. 3, *RRESEI,2A*). Detailed examination of the electron density maps in the later stages of refinement of RRESEI-PDZ1-2 revealed weaker electron density for the C-terminal residue of each ligand peptide, with negative difference electron density enveloping the terminal carboxylate in difference electron density maps. This was interpreted as an alternative conformation of this part of the bound ligand and was modeled with two alternative conformations of the C-terminal (−X_−1_-*Φ*_0_) -EI residues: one conformation has the Ile side chain buried in the binding cleft and the C-terminus of the peptide associating with the GLGF motif, as seen in the structures of other ligand-bound type I PDZ domains (shown for PDZ2 in Fig. 3, *RRESEI,2A*). The second conformation has the side chain of the Glu residue lying along the binding cleft, with the Ile side chain making an interaction with Ile 100 and Ile 195 in PDZ1 and PDZ2, respectively (shown for PDZ1 in Fig. 3, *RRESEI,1B*). This model maintains a carboxylate function close to the GLGF motif of each PDZ domain for each alternate −EI conformation while accounting for the observed features of the electron density. The model has a 0.5% lower R-free value (50) compared with a single RRESEI conformation model (with each Ile side chain buried in the cleft).

For apo-PDZ1-2, the structure shows a higher level of disorder overall (Fig. S8 *A*). The PDZ1 domain binding cleft is ordered, but the PDZ2 domain shows disorder in both the *β*A-*β*B loop and *α*A-helix regions (Fig. S8, *A* and *B*). Weaker and more diffuse electron density is encountered for these segments, which is consistent with disorder around the PDZ2 peptide binding cleft, including the GLGF motif (Fig. 3, *GLGF*). The *α*A-helix links directly to the GLGF motif in both domains (shown in Fig. 3, *GLGF-αA for PDZ1*). For PDZ2, the binding cleft is linked to the *α*A-helix through main-chain hydrogen bonds between Leu 170 O and Ala 200 (O-N distance of 2.9 Å) and Gly 171 and Ile 195 (O-N distance of 2.9 Å). The electron density of the apo-PDZ2-*β*A-*β*B loop can be fitted with a dual conformation (Fig. 3, *GLGF*; Fig. S8 *B*). The two conformers are 1) a conformation similar to the RRESEI-PDZ1-2 *β*A-*β*B loop and 2) a conformation similar to that found for the *β*A-*β*B loop of the syntrophin PDZ domain in the crystal structure of the neuronal nitric-oxide-synthase-syntrophin PDZ heterodimer (51) (PDB: 1qav). These two apo-PDZ2 *β*A-*β*B loop conformations (Fig. S8 *B*) were assigned the same occupancy, but after refinement, the residual B-factors were systematically lower for the RRESEI-PDZ1-2-like conformation, indicating a higher occupancy for this conformer.

In both apo-PDZ1-2 and RRESEI-PDZ1-2, an additional electron density distribution was found adjacent to residues F119 and Y147. In both crystal structures, the S-shaped electron density resembles a short peptide and has no connectivity linking it to the PDZ1-2 model. N- and C-terminal residues (GPNGT and SNA, respectively) from the tag-cleaved protein are not seen in the electron density maps. The location of the S-shaped electron density was distant from the C- or N-terminus of any PDZ1-2 model in the crystal lattice. The electron density could not be accounted for satisfactorily by fitting the PEG crystallization precipitant for either apo-PDZ1-2 or RRESEI-PDZ1-2. The cofactor-reduced GSH (L-*γ*-glutamyl-L-cysteinyl-glycine) was used in all but the final protein purification step at a millimolar concentration (in the lysis buffer; see Materials and Methods). A common association motif for GSH is the interaction of an amide plane from GSH with an aromatic residue side chain from the protein (for example, PDB: 5bqg (52)). The S-shaped electron density was fitted effectively with a single conformation of GSH with amide plane-aromatic stacking interaction with both F119 and Y147 (Fig. 3, *GSH*).

The differences between the individual PDZ1 and PDZ2 structures in apo-PDZ1-2 and RRESEI-PDZ1-2 are therefore limited to local changes in the vicinity of the peptide ligand binding site. The local disorder in apo-PDZ1-2 around the peptide binding site of PDZ2 and the *α*A-helix is not seen in RRESEI-PDZ1-2 when the RRESEI peptide occupies the binding site (Fig. S8 *A*). This shows that the peptide ligand binding site of PDZ2 is stabilized by the presence of the RRESEI ligand and that this stability is communicated to the *α*A-helix.

### ITC

The crystal structure of RRESEI-PDZ1-2 shows that similar noncovalent interactions are observed for RRESEI association with both PDZ1 and PDZ2 binding clefts. A comparison of the crystal structures of apo-PDZ1-2 and RRESEI-PDZ1-2 indicates an ordering of the GLGF loop and *α*A-helix regions of PDZ2 after RRESEI association. In contrast, the PDZ1 binding cleft is the same in both structures. To further inform the observed differences in the association of ligand with PDZ1 and PDZ2, the thermodynamic changes on association of separate PDZ1, PDZ2, and dual PDZ1-2 domains with RRESEI were each measured using ITC.

**Figure 4:**
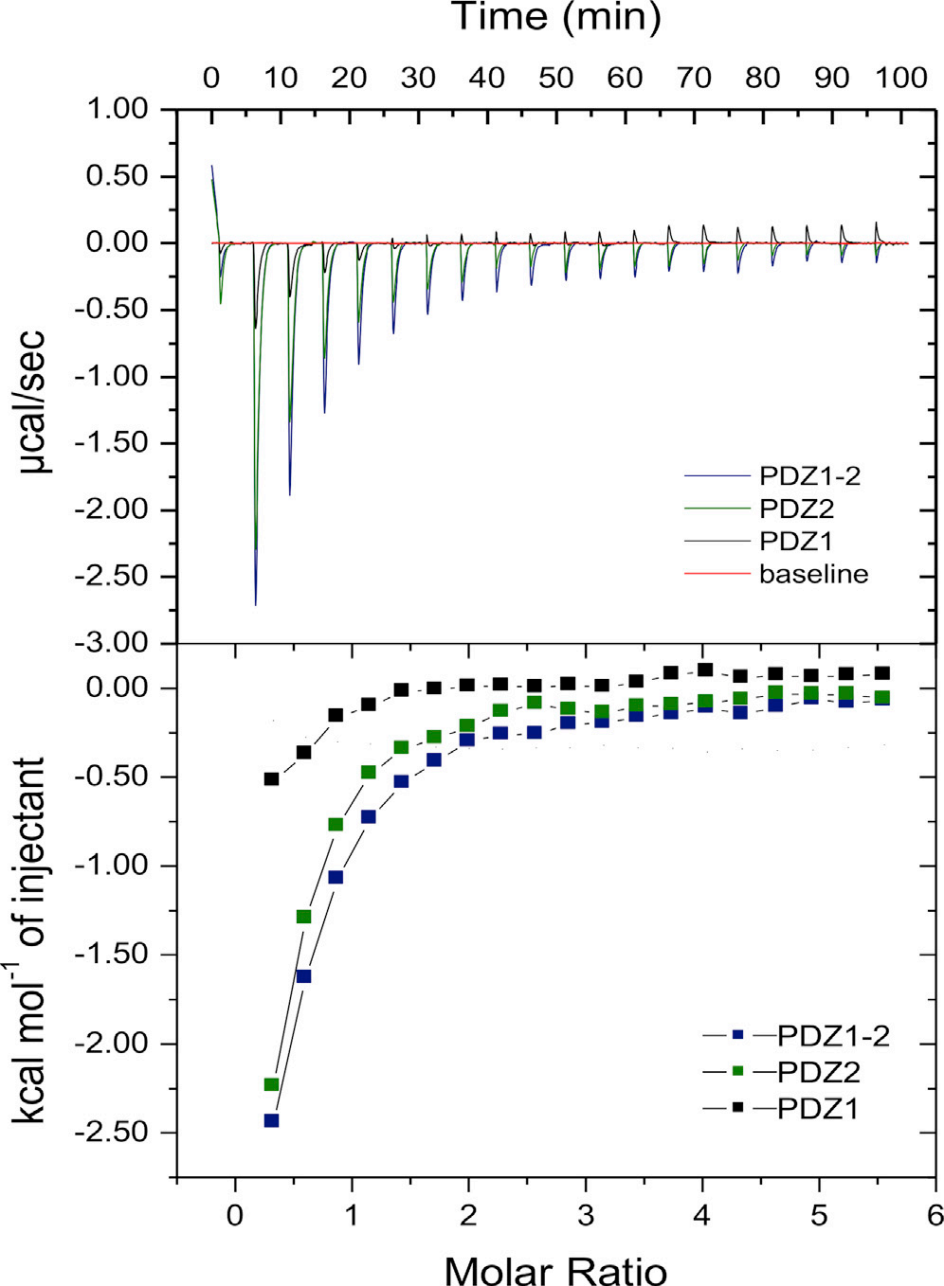
ITC of the association of PDZ1, PDZ2, and PDZ1-2 with the RRESEI ligand. ITC traces (*top*) and saturation analysis (*bottom*) for PDZ1, PDZ2, and PDZ1-2, each titrated with the RRESEI ligand, are shown overlaid. The heat change on successive injections of ligand is shown in the top panel buffer heat of dilution subtraction (baseline in *red*). The corresponding binding isotherm with fitted curve is shown in the bottom panel. To see this figure in color, go online.

All of the ITC data show that RRESEI binding to the macromolecule is saturable (Fig. 4). The form of each binding curve tends toward hyperbolic rather than sigmoidal, which is consistent with the relatively weak binding affinity (53). The equilibrium dissociation constant and binding stoichiometry (K_d_ and N), was derived from the ITC curve at 6 ± 6 and 0.61 ± 0.08 *μ*M and 60 ± 2 and 0.19 ± 0.06 *μ*M for PDZ1 and PDZ2, respectively. The K_d_-values obtained for the affinity between the individual PDZ domains and the RRESEI peptide are within the range of those found for similar peptides binding to PDZ domains in the literature (54). For the PDZ1-2 double domain, two individual affinities could not be distinguished from the ITC measurement, and overall values of K_d_ of 90 ± 9 *μ*M and N of 0.20 ± 0.07 *μ*M were obtained assuming a single class of sites. In ITC, the dissociation constant is derived from the midpoint of a curve fitted to the (sigmoidal) thermogram; hence, the values of K_d_ obtained for the binding of RRESEI to PDZ1, PDZ2, and PDZ1-2 are not well defined, and the stoichiometry N and K_d_ could not be refined independently in a stable way (53). The derived parameters (*Δ*G and *Δ*S) will therefore also be imprecise. Because the same buffer conditions were used for all experiments, protein concentrations were set at the same level, and the injected ligand concentration was the same, general comparisons between the form of the ITC characteristics can be made.

The thermogram of the PDZ1-2 and PDZ2 ITC experiment each showed exothermic peaks at every ligand injection. For the PDZ1 domain, the thermogram was more complex, showing exothermic binding initially, with a smaller endothermic binding change also apparent close to saturation (Fig. 4). Despite equivalent concentrations of the protein and ligand, the heat change at each injection is much smaller for PDZ1 compared with PDZ2 (Fig. 4).

In the transition from apo (free PDZ and free RRESEI) to bound (RRESEI-PDZ) states, a number of changes occur. The displacement of water from the binding cleft and the reduction in the degree of RRESEI structural freedom would appear to be similar for both PDZ1 and PDZ2 binding sites. The formation of noncovalent interactions between RRESEI and the PDZ binding site are also similar for both PDZ1 and PDZ2 sites, as seen in the RRESEI-PDZ1-2 crystal structure. An ordering of the PDZ2 domain binding cleft on interaction with the peptide is seen when comparing the apo and RRESEI-PDZ1-2 structures. The disordered *β*A-*β*B loop of apo-PDZ1-2 assumes a single conformation in RRESEI-PDZ1-2, and the PDZ2 *α*A-helix has a lower temperature factor (Fig. S8). Such an increase in the overall order of the bound complex would imply a negative *Δ*S contribution, leading, in turn, to a higher *Δ*G and weaker binding. This may, therefore, be one source of the difference in the ITC measurements between RRESEI binding to PDZ1 and PDZ2 but does not account for the complex form of the binding isotherm for RRESEI association with PDZ1.

The additional endothermic contribution seen in the thermogram of PDZ1 is systematically associated with each ligand injection but occurs over a longer timescale. An underlying endothermic contribution would in part reduce the exothermic heat change at each ligand injection. Relative to RRESEI binding to PDZ2, the gross effect of the additional endothermic contribution seems to be a foreshortening of the saturation characteristic. The difference in calculated affinity between PDZ1 and PDZ2 may therefore also be linked to the endothermic element seen in the PDZ1 titration.

### SAXS

The single conformation of PDZ1-2 in the crystal structures reported here accommodates binding of the RRESEI peptide ligand to both PDZ1 and PDZ2 domains. This PDZ1-2 conformation is different from those seen in the earlier PDZ1-2 crystal structures (Fig. S9). The gross form of PDZ1-2 is therefore variable and may depend upon the interaction with the peptide ligand. To explore this variation, the PDZ1-2 domain was examined in solution using SAXS in both the apo state and in the presence of a 10-fold molar excess of RRESEI. GSH was assigned in the crystal structures. GSH is present in the cell, and GSH possesses both thiol and carboxylate functions in common with type I PDZ ligand sequences. GSH association with PDZ1-2 was also explored using SAXS. For each set of SAXS measurements, matching scattering data on a sample of apo-PDZ1-2 were collected. All data sets are summarized in Table S2.

For the fractionated SAXS experiments, an absorption chromatogram at a wavelength of 280 nm was recorded immediately before exposure with x rays. A scattering profile was then recorded for one or more fractions. In contrast to the preparative SEC step, a broader highly absorbing peak was observed in the 280-nm chromatograms preceded by a small peak or shoulder. The precise form of this main peak varied between sample injections, with a flat top, unresolved doublet, or a single peak with a shoulder being observed. Where R_g_ analysis was carried out for multiple fractions (June 2016 data), the obtained values of R_g_ showed no significant variation across the main peak.

The apo-PDZ1-2 and RRESEI-PDZ1-2 data showed evidence of both interparticle association effects and underlying multiple conformations of PDZ1-2 (see Materials and Methods). The level of these two features differed according to both the concentration of the PDZ1-2 protein and the presence of the RRESEI peptide ligand.

### Oligomer model construction

Crystal contacts and PDZ1-2 conformations drawn from existing crystal structures were used to generate oligomers for fitting the SAXS data (see Materials and Methods). The rationale for this approach was that at the ultrahigh protein concentrations represented in protein crystals, intermolecular interactions would appear as crystal contacts. There are a finite number of ways that single molecules can arrange themselves in extended repeating arrays in three dimensions (48). Therefore, the assignment of a space group to the oligomeric structures encountered for PDZ1-2 was investigated. A cubic I2_1_3 space group with a unit cell parameter |**a**| = 148 Å could be assigned to the oligomers of PDZ1-2 in an unambiguous manner (see Materials and Methods). Various oligomers can be reproduced by selecting the appropriate combinations of symmetry operations from this I2_1_3 “scaffolding space group.” An additional PDZ1-PDZ1 interaction predicted by the molecular packing in the assigned I2_1_3 space group is present in a number of these oligomers.

### Order of oligomer assembly

The formation of any protein-protein complex is governed by the abundance of the assembling components and the affinity of the components for one another. In the scaffolding space group, each PDZ1-2 monomer makes two interactions of the form *α*B(PDZ2)-*β*D-*β*E(PDZ1), two of *α*A(PDZ2)-*β*B-*β*C(PDZ1), and one PDZ1-PDZ1 interaction. There is no reliable independent measurement of affinity for these three individual interactions. Estimates of affinity can be derived from a computational analysis of the interfaces and compared. The *α*A(PDZ2)-*β*B-*β*C(PDZ1) interaction is seen in the 3gsl-PDZ1-2 structure and using the Proteins, Interfaces, Structure, and Assemblies (PISA) server (55); a *Δ*^i^G-value of −9.2 kJ/mol is assigned for *α*A(PDZ2)-*β*B-*β*C(PDZ1). In 3zrt-PDZ1-2, the dimer formed by two copies of PDZ1-2 is seen for chains C and A. This interaction is assigned a *Δ*^i^G-value of −22.2 kJ/mol by PISA and includes two *α*B(PDZ2)-*β*D-*β*E(PDZ1) interfaces plus the interaction between residues in the two copies of the *β*B-*β*C(PDZ2) loop. The interactions are all noncovalent, with hydrogen bonding and ion pair interactions dominating each interface. For the analysis of PDZ1-2 assembly into oligomers, it was assumed that the affinity of each of the individual interactions (*α*B(PDZ2)-*β*D-*β*E(PDZ1), *α*A(PDZ2)-*β*B-*β*C(PDZ1), and PDZ1-PDZ1) is similar in magnitude. The order of assembly as the overall concentration of PDZ1-2 increases is then determined via the principle of avidity: a metastable complex occurs when multiple (avid) interactions form between binding partners because of the factorial increase in affinity. In other words, interactions at two or more separate sites would need to be broken at the same time for a complex so formed to dissociate.

**Figure 5:**
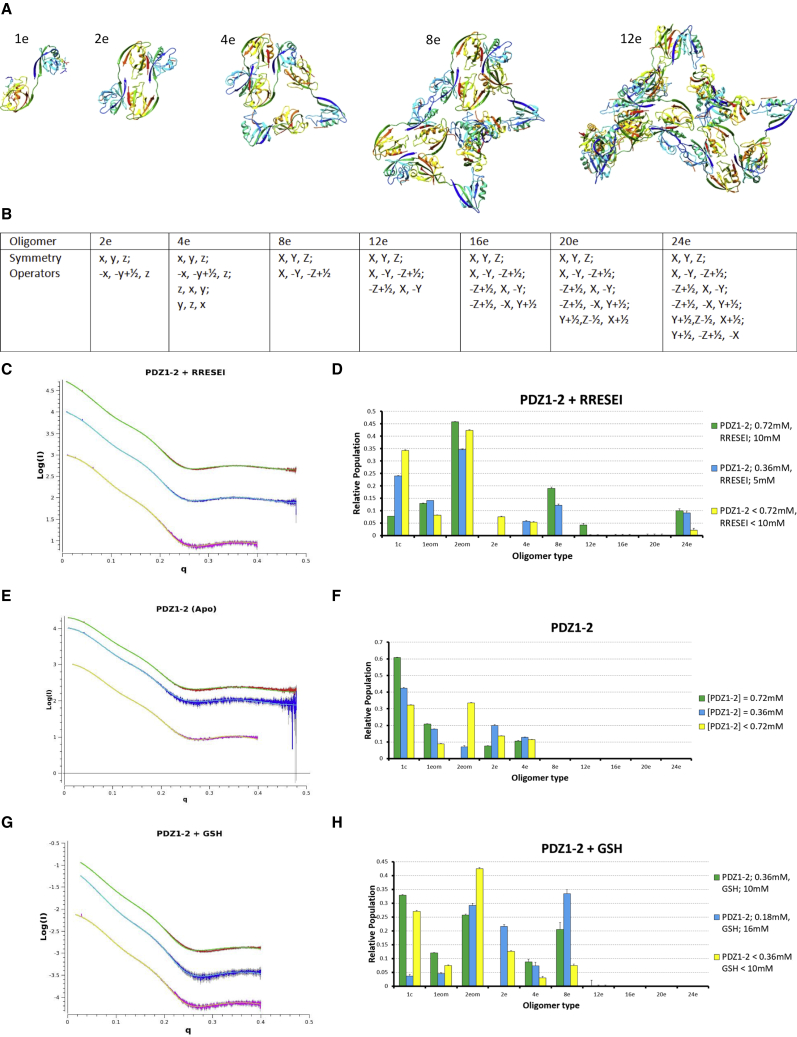
Oligomer fitting of SAXS data. Selected PDZ1-2 oligomers 1e, 2e, 4e, 8e, and12e are shown in (*A*), where “e” denotes the extended 3zrt-like PDZ1-2 conformation; the representation is similar to Fig. 1. The symmetry operations drawn from the scaffolding space group required for the construction of the PDZ1-2 oligomers used in fitting are given in (*B*). Transformations applied to the fractional coordinates of the PDZ1-2 monomer (*A*, *1e*) are given in lower case (*x*, *y*, and *z*) and those applied to the metastable tetramer (*A*, *4e*) in capital letters. Fitted SAXS curves are shown alongside histograms of the oligomer populations in (*C*)–(*H*) for RRESEI, apo, and GSH-PDZ1-2 in turn. Scattering curves are plotted as Log(I) versus q throughout, where I is the scattering intensity and q is momentum transfer in Å^-1^ (Log(I)- versus Log(q)-transformed plots are also shown in Fig. S11). High-concentration data curves are shown in red with fitted curves in green, dilutions in blue with a fitted curve in cyan, and SEC-fractionated samples in magenta with fitted curves in yellow. A multiplication factor has been applied to the raw data in some cases to separate curves along the ordinate (Log(I)) axis. The histograms show the relative populations of various oligomers determined in the fitting analysis. The populations were determined by multiplying the volume fraction assigned by the OLIGOMER program (41,47) by the oligomer number and then renormalizing all fractions to sum to 1, the error bars were propagated from the error estimates given by OLiGOMER. Hence, the columns show the proportion of PDZ1-2 molecules assigned to each oligomer. Shown are the “1c” oligomer shown on the abscissa maps to compact PDZ1-2 (Fig. 3, *A* and *B*) and the two “eom” oligomers to uncoupled versions of PDZ1-2 derived from the analysis of RRESEI-PDZ1-2 with the EOM program (45) (the eom1 and 2 structures are shown in Fig. S10). To see this figure in color, go online.

A PDZ1-2 dimer may form through any one of the *α*B(PDZ2)-*β*D-*β*E(PDZ1), *α*A(PDZ2)-*β*B-*β*C(PDZ1), or PDZ1-PDZ1 interactions, with few restrictions on the gross conformation of PDZ1-2. If a 3zrt-PDZ1-2-like conformation (Fig. 5 *A*, *1e*) is adopted by the binding partners, two *α*B(PDZ2)-*β*D-*β*E(PDZ1) interactions form at the same time (Fig. 5 *A*, *2e*). This “double” dimer would therefore be metastable over the other pairwise interactions because of the avidity. This dimer and monomer mixture can be refined against SAXS data, with good agreement achieved for lower concentrations of PDZ1-2 (SEC-SAXS or data extrapolated to zero concentration).

Proceeding from this metastable dimer, a further complex could be formed by either an *α*A(PDZ2)-*β*B-*β*C(PDZ1) or a PDZ1-PDZ1 interaction. Either a dimer-monomer interaction or a dimer-dimer interaction would be possible, and the affinity of these are assumed to be the same. A dimer-monomer complex would be favored initially until the concentration of dimers exceeded the concentration of monomers. There would be few restrictions on the conformation of the associating monomeric PDZ1-2.

The addition of another monomer to this dimer-monomer complex to form a tetramer could again occur through any of the three interactions. In one particular case in which the preceding dimer-monomer interface was formed via *α*A(PDZ2)-*β*B-*β*C(PDZ1) interaction, a further monomer may add, forming two *α*A(PDZ2)-*β*B-*β*C(PDZ1) interactions, again subject to the adoption of a 3zrt-PDZ1-2-like conformation for all PDZ1-2 copies. This tetramer complex (Fig. 5 *A*, *4e*) would be metastable because in the oligomer, each PDZ1-2 is involved in at least two interactions. The metastable tetramer complex has three copies of PDZ1-2 related by a threefold rotation axis and two copies related by a twofold rotation axis. This configuration can be refined against SAXS data as a mixture with the PDZ1-2 monomer, and good agreement is achieved for lower PDZ1-2 concentrations in which the RRESEI ligand is present. The angle between these threefold and twofold symmetry axes is variable in the resulting refined models, with a larger angle (∼90°) seen for refinement with data collected on more dilute samples. A consequence of the large angle between the oligomer symmetry elements is that the addition of more monomers to the tetramer via *α*B(PDZ2)-*β*D-*β*E(PDZ1) interactions would be sterically hindered.

In the scaffolding space group, the angle between the threefold and twofold symmetry elements (as in Fig. 5 *A*, *4e*) is fixed at 54.7°. Thus, a particular conformation of the metastable tetramer is required for assembly into even higher order structures in a similar way to the requirement of the monomeric form of PDZ1-2 to adopt the 3zrt-PDZ1-2 conformation for complex formation. When the scaffolding space group tetramer configuration is used, two tetramers can associate to form an octamer by forming two *α*B(PDZ2)-*β*D-*β*E(PDZ1) interactions. In one of the three possible configurations of this octamer, an additional PDZ1-PDZ1 contact is also made. This particular configuration would therefore be favored as three interactions are formed on assembly (Fig. 5 *A*, *8e*). Subsequent additions of tetramers, each making three interactions, may then be made for 12- (Fig. 5 *A*, *12e*), 16-, 20-, and 24-mer oligomers, the latter corresponding to the unique components of the unit cell of the (body-centered) scaffolding space group.

### SAXS data fitting

The initial analysis of the SAXS data outlined in the Materials and Methods established that fitting requires a model that describes both the oligomers formed by PDZ1-2 and the variation in the structure of an isolated PDZ1-2 monomer. PDZ1-2 monomer variation is efficiently described by the compact conformation of PDZ1-2 from the crystal structure reported here plus two models from one instance of the EOM analysis of scattering data projected to infinite dilution. The EOM-derived monomers were similar in form to structures found in 3gsl-PDZ1-2 or 2ka9-PDZ1-2, having no direct noncovalent intramolecular PDZ1-PDZ2 contacts (Fig. S10).

The I2_1_3 clustering space group defines the unique fraction of a repeating lattice. The oligomer components are therefore limited primarily by the number of general equivalent positions in the space group. Even within this limitation, up to a theoretical limit of 24! (∼6.2 × 10^23^) combinations of these monomers from the clustering space group could be included in fitting. The least-biased approach to oligomer fitting would be to sort these combinations into the unique set of combinations that have mutual contacts and subsequently use a principle component analysis to determine the minimal set required to fit each curve. Such an approach would require considerable logical and computational analysis. The approach used here was to use the seven oligomers drawn from the order of assembly analysis outlined above in oligomer-based fitting. This approach is also systematic and serves the dual purpose of limiting the number of model parameters used to describe the data, along with adding interpretation regarding the formation of stable intermediates. The methodology deployed to interpret the scattering data is compelling because relatively few degrees of freedom are required to describe the complete structural model, and a variation of oligomer populations with PDZ1-2 concentration is readily accounted for.

Data fitting with OLIGOMER (Fig. 5, *C*–*H*) was carried out using the metastable complexes described above (monomer, dimer, tetramer, octamer, and 12-, 16-, 20-, and 24-mer oligomers). The relative proportions of PDZ1-2 monomers assigned to the various oligomers for apo-PDZ1-2 and RRESEI-PDZ1-2 data are shown in Fig. 5, *D* and *F*. In the case of apo-PDZ1-2, monomer, dimer, and tetramer oligomers are sufficient to account for the scattering (Fig. 5 *F*). In the case of RRESEI-PDZ1-2, oligomers extending up to a 24-mer are needed to account for the observed scattering profile (Fig. 5 *D*). Some marginal improvements in the agreement of the curve with the data were observed if larger oligomers were included. However, the inclusion of oligomers extending beyond one repeating unit would imply some interference in the scattering; because this may break assumptions inherent in the methodology used to generate the calculated SAXS curves that are fitted, larger oligomers were not included.

The scattering data collected in the presence of GSH (GSH-PDZ1-2) were fitted through direct application of the same methodology. The GSH-PDZ1-2 data require fewer oligomer classes, with good agreement for oligomers of up to eight copies of GSH-PDZ1-2 (Fig. 5 *H*). In the case of GSH-PDZ1-2, the relative concentration of GSH and PDZ1-2 vary for each scattering experiment (see Materials and Methods). Here, the higher-order oligomers are associated with the highest GSH/PDZ1-2 ratio (Fig. 5 *H*).

For the oligomer models of RRESEI-PDZ1-2, Χ^2^-values of 8.74, 2.71, and 3.71 were obtained for concentrated, diluted, and SEC data, respectively; the equivalent values for apo-PDZ1-2 are 6.31, 1.62, and 2.67 and for GSH-PDZ1-2 are 1.41, 0.33, and 4.21. The agreement of the oligomer models therefore compares well with the dummy atom modeling with data projected to infinite dilution (see Materials and Methods). The oligomer-based fitting is an improvement over the dummy atom fitting because scattering data over the complete momentum transfer interval recorded are included (Fig. 5, *C*, *E*, and *G*; the quality of fitting in the higher q resolution range is also shown by the Log(I) versus Log(q) plots shown in Fig. S11).

As mentioned in the Materials and Methods, in the case of unfractionated RRESEI-PDZ1-2, a contribution from the free ligand is present in the scattering data. CRYSOL (56) calculations based upon the RRESEI models extracted from the crystal structures reported here indicate that the scattering contribution is of the form of a low level and smoothly varying I(q) function over the range 0 < q < 0.5 Å. A scattering component for a free RRESEI peptide can be included in the model-based analysis. The contribution from free RRESEI corresponds to a volume fraction of 0.051 ± 0.001 for the concentrated RRESEI-PDZ1-2 data with a decrease in Χ^2^-value (7.0 compared with 8.7 for fitting the concentrated RRESEI-PDZ1-2 SAXS curve).

For all of the SAXS experiments reported here, lower concentrations of PDZ1-2 derive from a higher concentration sample either through dilution or for the fractionated SAXS data via diffusion during passage down a size-exclusion column (see Materials and Methods). We assume that the timescales of the biophysical experiments here are long enough such that equilibrium conditions pertain. When comparing oligomer populations for 0.72 and 0.36 mM and SEC-SAXS data for RRSEI-PDZ1-2 in Fig. 5 D, it can be seen that larger oligomers persist after dilution. This feature of the oligomer models serves to account for the apparent anomalies in the I(0)-values observed in the SAXS data and noted in the Materials and Methods. This effect may therefore reflect a difference in the assembly (the progressive association to form larger components) over disassembly (dissociation into smaller components) of the oligomers. This hysteresis appears to go against our expectation because the individual interfaces are formed by noncovalent associations. However, when considering the stepwise breakdown of particular oligomers, the basis underlying this effect is clearer. The dissociation of a PDZ1-2 monomer from complexes 2e-4e shown in Fig. 5 *A* requires two PDZ1-2-PDZ1-2 interactions to be broken. For a fully formed I2_1_3 scaffolding lattice, the dissociation of a PDZ1-2 monomer requires five PDZ1-2-PDZ1-2 interactions to be broken. This means that complexes between these limiting conditions (for example, those shown in Fig. 5 *A8e* and *12e*) become progressively more stable with the number of participant units and are more likely to remain after dilution.

### Compatibility of GSH and RRESEI ligands with the oligomer interfaces

An analysis of the PDZ1-2 structures reported here using PISA (55) indicates that the binding affinity of GSH to the site at F118-Y147 on the surface of PDZ1 is slightly lower (*Δ*^i^G = −12.6 kJ/mol) than the affinity of RRESEI to the binding cleft of PDZ1 or PDZ2 (*Δ*^i^G = −14.2 and −13.8 kJ/mol, respectively). The estimated K_d_ based upon this would therefore be greater by a factor of 2.4. The PISA *Δ*^i^G estimate is based upon the difference in solvation in the bound and unbound states. The resulting difference in calculated free energy is due to the buried surface area. This change has a greater net thermodynamic effect compared with the exchange of hydrogen bonds with solvent for those to protein. The *α*B(PDZ2)-*β*D-*β*E(PDZ1) interface, as seen in the 3gsl-PDZ1-2 structure, includes both F118 and Y147, with the latter forming a hydrogen bond with H225 of PDZ2. If GSH is present, the F118/Y147 side chains are in contact with the amide planes of GSH (Fig. 3, *GSH*), and GSH moieties such as the thiol and carboxylate will be presented to the PDZ2 binding cleft. The thiol carboxylate and amide groups from GSH can then, in turn, participate in interactions with the PDZ2 cleft on formation of the *α*B(PDZ2)-*β*D-*β*E(PDZ1) interface. It is likely, therefore, that GSH effectively modifies the surface of PDZ1 such that an *α*B(PDZ2)-*β*D-*β*E(PDZ1) interface is enhanced.

The RRESEI ligand can be accommodated within the *α*B(PDZ2)-*β*D-*β*E(PDZ1) interface after the adjustment of side-chain rotamers of aromatic residues Y63 and Y147 of PDZ1-2. The ligand peptide may then participate in a continuous *β*-sheet with RRESEI intercalated between the *β*A-strand of PDZ1 (forming parallel *β*-sheet interactions) and the *β*B-strand of PDZ2 (forming antiparallel *β*-sheet interactions). The alternative side-chain rotamer for Y147 changes the configuration of the PDZ1 surface in the region of the observed GSH site. Additionally, although either RRESEI or GSH can be accommodated in the PDZ2 binding cleft enclosed by the *α*B(PDZ2)-*β*D-*β*E(PDZ1) interface, both peptides could not simultaneously bind without stereochemical interference. Thus, the weaker-binding GSH would likely be excluded from the interface if a peptide ligand were present in the PDZ2 cleft. Therefore, both RRESEI and GSH could knit together the *α*B(PDZ2)-*β*D-*β*E(PDZ1) interface, but the inclusion of RRESEI or GSH in the *α*B(PDZ2)-*β*D-*β*E(PDZ1) interface is mutually exclusive.

The other contact used in defining the I2_1_3 scaffolding lattice is *α*A(PDZ2)-*β*B-*β*C(PDZ1). There is no direct involvement of binding clefts in this interface. The relationship between the stability of the *α*A-region in PDZ2 and the association of RRESEI at the PDZ2 cleft (Fig. S8) indicates that ligand recognition may be an indirect factor in the formation of this contact.

A direct interface between copies of PDZ1 is predicted within the I2_1_3 scaffolding lattice. An interface of this type can form between isolated PDZ1 domains. The alternative orientation of RRESEI with the C-terminal emergent from the PDZ1 ligand binding cleft seen in refinement of the RRESEI-PDZ1-2 crystal structure (Fig. 3, *RRESEI,1*B) appears to be compatible with this interface in the scaffolding space group. When this orientation is present, additional interactions are made at the interface, including the insertion of Asn 72 between the bound ligand and *α*B-helix of PDZ1. However, because the scaffolding arrangement is effectively determined by SAXS analysis, which is limited to a lower resolution, structural details of this type should be treated with caution.

### Influence of RRESEI and GSH on oligomer formation

The K_d_-values observed in the ITC analysis of PDZ1-2-RRESEI binding are not simply related to their counterparts extracted for isolated domains. This suggests that either binding site interference (in which binding at one domain blocks binding at the second domain) or interdomain interactions may be at play. The crystal structures of PDZ1-2 do not support any mechanism of direct site interference for a short peptide like RRESEI, whereas the SAXS measurements show that RRESEI enhances the formation of PDZ1-2 oligomers.

The association of RRESEI with PDZ1-2 is correlated with a lower population of the compact form of PDZ1-2 (seen when comparing populations of *1c* in Fig. 5, *D* and *F*). The transition from compact to more extended PDZ1-2 increases the number of conformations that can be adopted by PDZ1-2 alongside the replacement of an intramolecular interaction (Fig. 3, *αA-βC-βD*) with a solvent interaction. The increased number of gross conformational states would lead to an increase in entropy, and the exchange of interactions will lead to a change in enthalpy. According to the SAXS analysis presented here, oligomers containing the extended conformation (Fig. 5 *A*, *1e*) form in both apo- and RRESEI-bound cases, with higher levels seen in the RRESEI-bound case (Fig. 5, *D* and *F*). The formation of oligomers constrains the conformation of PDZ1-2 alongside the formation of noncovalent interactions between PDZ1-2 copies. The additional factors leading to oligomerization therefore make diverse thermodynamic contributions. These additional interactions could readily account for the differences between the separate PDZ domains and the double domain seen in the ITC titrations with RRESEI.

The SAXS analysis indicated that the formation of the PDZ1-PDZ1 interface is important for larger oligomers like those required for fitting RRESEI-PDZ1-2, in turn suggesting that the binding of RRESEI enhances the formation of this interface. This would explain the unusual behavior of the PDZ1 domain in ITC in which an additional endothermic heat change is resolved close to saturation with the RRESEI ligand, the additional heat change being due to the association of RRESEI-PDZ1 domains with one another. In the ITC experiment, the PDZ1 domain is successively diluted in the measurement cell by the addition of aliquots of the RRESEI solution. Thus, any association of PDZ1 domains must be RRESEI mediated. Interfaces between copies of peptide-bound PDZ domains have been seen in crystal structures, for example, PDB: 1oby (57).

PDZ1-2 oligomers in the presence of GSH alone are more limited in extent than those in the presence of RRESEI. The structural data indicate the influence of GSH is primarily in the *α*B(PDZ2)-*β*D-*β*E(PDZ1) interface. Using the scaffolding space group, it is possible to devise octamer and dodecamer assemblies of PDZ1-2 tetramers that do not have a PDZ1-PDZ1 interaction (a dodecamer can be formed from tetramer symmetry operations, in similar manner to Fig. 5 *B*, *X*, *Y*, and *Z*; *Y*, *−Z*, and *−X+1/2*; and *−X+1/2*, *Y*, and *−Z*). The shorter GSH peptide ligand may not significantly enhance the PDZ1-PDZ1 interface predicted in the scaffolding space group, and this may explain why oligomers are more limited in extent in the presence of GSH.

Despite the presence of the PDZ1 domain in PDZ1-2, the endothermic peaks observed near saturation for PDZ1 are not seen in ITC titrations of PDZ1-2 with RRESEI. There is a systematic difference between the preparation of PDZ1-2 and of individual domains for ITC. GSH was not used in the purification of individual PDZ domains, but GSH is likely to be present in the PDZ1-2 purified for ITC (carried over as in the case of crystallized PDZ1-2). In this case, GSH-PDZ1-2 association would decrease in the ITC cell during the titration and association between PDZ1-2 could be promoted by RRESEI. There may, therefore, be an interplay between the three factors of GSH and RRESEI binding and oligomer formation when neither of the peptide ligands are in excess over PDZ1-2.

## Discussion

The preparation of subcellular neuronal compartments such as PSDs for imaging or extraction of proteins often necessitates harsh treatments such as mechanical disruption, multiple centrifugation steps, and extensive dilution (58). A recent report also indicates that detergent-specific effects can occur when isolating component oligomers from PSD fractions (59). The noncovalent interactions analyzed here are likely to be compromised in subneuronal isolates; therefore, the approach of assembling component elements taken in this study has the power to reveal new, to our knowledge, information. In their dissection of the clustering properties of PSD-95, Hsueh et al. noted that, “A rafting mechanism for clustering of multimeric channels … could still be accomplished by a single PDZ domain per PSD-95 monomer if PSD-95 self-associates to form multimers.” The work here shows that extensive association of PSD-95 can occur, but this is acutely sensitive to the binding ligand. Because the association is driven by relatively weak noncovalent interactions, a high concentration of PSD-95 is also required. It has been shown in vitro that a trimer of the C-terminal portion of the synaptic RAS GTPase-activating protein (SynGAP; including a C-terminal QTRV motif) and PDZ3-SH3GK spontaneously form a condensed solution phase at high concentrations (21). Subsequently, it was shown that a cohort of four PSD scaffold proteins (full-length PSD-95, Homer3, and interacting fragments of GKAP and Shank) can recruit a chimeric tetramer representative of NMDAR to a protein-enriched phase via binding to PDZ1-2 of PSD-95 (22). The latter condensation is observed as a cluster formation in a model bilayer system, and SynGAP was shown to be nonessential for this cluster formation. The PDZ1-2 and PDZ3-SH3GK elements of PSD-95 therefore appear to have distinct roles in scaffolding function. The results reported are consistent with a scaffold containing PSD-95 linking the receptor-bearing synaptic surface (binding at PDZ1-2) with the regulatory SynGAP protein (binding at PDZ3).

### The role of the peptide ligand

A significant feature of the apo-PDZ1-2 crystal structures reported here is the disorder of the PDZ2 cleft and the PDZ2 *α*A-helix (Fig. 3, *GLGF*; Fig. S8) in contrast to 3gls-PDZ1-2, in which the vacant PDZ2 ligand binding site is ordered for both copies of PDZ1-2 (this is also true of 3zrt-PDZ1-2, although lower resolution is a factor for this structure). In both 3gls-PDZ1-2 and 3zrt-PDZ1-2, the binding cleft of each PDZ2 domain is involved in the *α*B(PDZ2)-*β*D-*β*E(PDZ1) interaction with a PDZ1 domain from another PDZ1-2. This *α*B(PDZ2)-*β*D-*β*E(PDZ1) intermolecular contact may therefore stabilize the conformation of the vacant PDZ2 ligand binding site. In 3gls-PDZ1-2, intermolecular contacts between Tyr 147 and His 225 and Glu 65 and Ser 173 are formed at this interface. In 3gls-PDZ1-2, the *α*A-helix of PDZ2 is additionally stabilized by a crystal contact *α*A(PDZ2)-*β*B-*β*C(PDZ1) with a second PDZ1-2 molecule. The *α*B(PDZ2)-*β*D-*β*E(PDZ1) and *α*A(PDZ2)-*β*B-*β*C(PDZ1) can therefore form in the absence of ligands.

The two bound RRESEI peptides in the RRESEI-PDZ1-2 structure show similar features, including weaker electron density at their C-terminus, which is interpreted here with a dual conformation of the terminal EI residues of both of the bound peptide ligands. The disorder of the C-terminal residues of the RRESEI ligand in the PDZ1 cleft seen in RRESEI-PDZ1-2 would appear to be an unexpected result given the ordered nature of the unoccupied PDZ1 site in apo-PDZ1-2. The alternate bound conformation of RRESEI lacks the hydrophobic group inserted into the cleft but forms an interaction with a hydrophobic residue in a loop immediately preceding the *α*A-helix (Fig. 3, *RRESEI,1*B).

The stability of the PDZ2 peptide ligand cleft is enhanced when intermolecular contacts are made between the cleft or the adjacent *α*A-helix or when a peptide ligand is bound. This indicates that there is likely to be a synergy between the conformation of the PDZ2-cleft-PDZ2-*α*A-helix and the presence of the peptide ligand. This can be mediated by the GLGF sequence, which links these two substructures via noncovalent interactions (as in shown Fig. 3, GLGF-*α*A).

The gross structures of the two 3gsl-PDZ1-2 are less compact than the structures reported here because the intramolecular *α*A(PDZ1)-*β*B-*β*C(PDZ2) interaction is not formed (Fig. S9). In the 3gsl crystal structure, the −ETMA ligand and the PDZ1 cleft are ordered, and the Met residue forms a hydrophobic contact with Ile 100 on the *β*C-*α*A loop. A comparison with the RRESEI-PDZ1-2 raises the possibility that a synergy between the binding cleft and the *α*A-helix of PDZ1 also exists. The ligand peptide may induce a conformational change in the peptide binding cleft, which is relayed to the *α*A-helix by the GLGF region and a hydrophobic contact between the ligand and Ile 100 (Fig. 3, GLGF-*α*A). This local conformational change, in turn, decouples any *α*A(PDZ1)-*β*B-*β*C(PDZ2) intramolecular interaction (Fig. 3, *αA-βC-βD*).

Taken together, these facets indicate that a subtle induced fit mechanism may be at work for the interaction of RRESEI with PDZ1-2. RRESEI interaction at PDZ1 induces conformational changes, which, in turn, are relayed to the *α*A-helix of PDZ1. An uncoupled conformation of PDZ1-2 results from the dissociation of the intramolecular interaction. In contrast, RRESEI peptide interaction at PDZ2 induces a single ordered conformation of the GLGF-bearing cleft, which, in turn, is relayed to the *α*A-helix of PDZ2, favoring the ordered conformation of this helix seen in RRESEI-PDZ1-2. This ordered conformation is then able to form the intermolecular *α*A(PDZ2)-*β*B-*β*C(PDZ1) interaction seen in the scaffolding space group.

### The role of the conformation of PDZ1-2

The gross form of the PDZ1-2 domain is like a “dumbbell,” and two peaks consistent with this structure are clearly evident in the pair distance distributions derived from the scattering curves (Fig. S4). Underlying this overall form were systematic differences between both the scattering curves and their corresponding transformed pair distance distribution functions according to the concentration of PDZ1-2 and the presence of peptide ligands. Pair distributions of PDZ1-2 in complex with a peptide-based inhibitor of PSD-95 clustering also showed a similar form (25). The pair distribution function of PDZ1-2 in the presence of a monomeric inhibitor shown for this work appears to show a sharper shoulder peak in P(r), which is qualitatively similar to the form of P(r) encountered in the RRESEI:PDZ1-2 case here (Fig. S4). Some variation in the intradomain separation of PDZ1 and PDZ2 has also been reported in NMR studies (24), and domain separation was enhanced in the presence of a ligand.

The population of the compact form of PDZ1-2 is low for the higher concentration of RRESEI-PDZ1-2, as shown by the SAXS analysis (Fig. 5 *D*), with a small population of the compact form seen only for diluted RRESEI-PDZ1-2. Thus, the RRESEI-PDZ1-2 crystal structure described here represents a minority conformer in solution. This may explain the requirement for seeding in the formation of RRESEI-PDZ1-2 crystals (the seeds promoting nucleation at lower RRESEI-PDZ1-2 concentration in which there is a significant level of the compact monomeric form). In contrast in the apo case, the compact form of PDZ1-2 is present at an increased concentration, and the proportion of PDZ1-2 oligomers is lower (Fig. 5 *F*). This indicates a preference for the decoupled and extended conformation in the presence of a bound ligand. A synergy between the association of RRESEI peptide at the PDZ1 binding cleft, the conformation of the *α*A-helix of PDZ1, and the gross conformation of PDZ1-2 is a likely reason for this.

A major determinant in the formation of the I2_1_3 scaffolding lattice revealed here is the gross conformation of the PDZ1-2 domain. The oligomers, which, in turn, lead to the cubic packing arrangement, require the PDZ1-2 domain to adopt an extended (3zrt-PDZ1-2-like) form. A first step in the establishment of this extended conformation is the decoupling of the compact form of PDZ1-2. The ligand incorporated at the *α*B(PDZ2)-*β*D-*β*E(PDZ1) interface may change the affinity of this interdomain interaction. The properties of residues “X” in the sequence −X_−3_−(Ser-Thr-Cys)_-2_-X_−1_-*Φ*_0_ from the ligating partner may therefore modulate the affinity of PDZ-PDZ scaffolding interactions. In the work described here, the presence of a Glu residue at X_−1_ appears to be important in allowing an alternate peptide ligand conformation. The type of aliphatic side chain at *Φ*_0_ may, in turn, have an influence on either the perturbation of the PDZ ligand binding cleft when in a buried conformation or the formation of a contact with a residue on the *β*C-*α*A loop when in an alternate conformation. The NMDA receptor present in the PSD is assembled from combinations of four *ε*- and *ζ*-subunits (60); the *ε*-subunits have a C-terminal sequence of −E_−3_-S_−2_-(D/E)_−1_-V_0_. Hence, the NMDA receptor also possesses carboxylic-acid-bearing residues at the X_−1_ position, and the sequence will have similar effects on PDZ1-2 as RRESEI.

### The PDZ1-2 domain can form the core of a MAGUK scaffold

**Figure 6:**
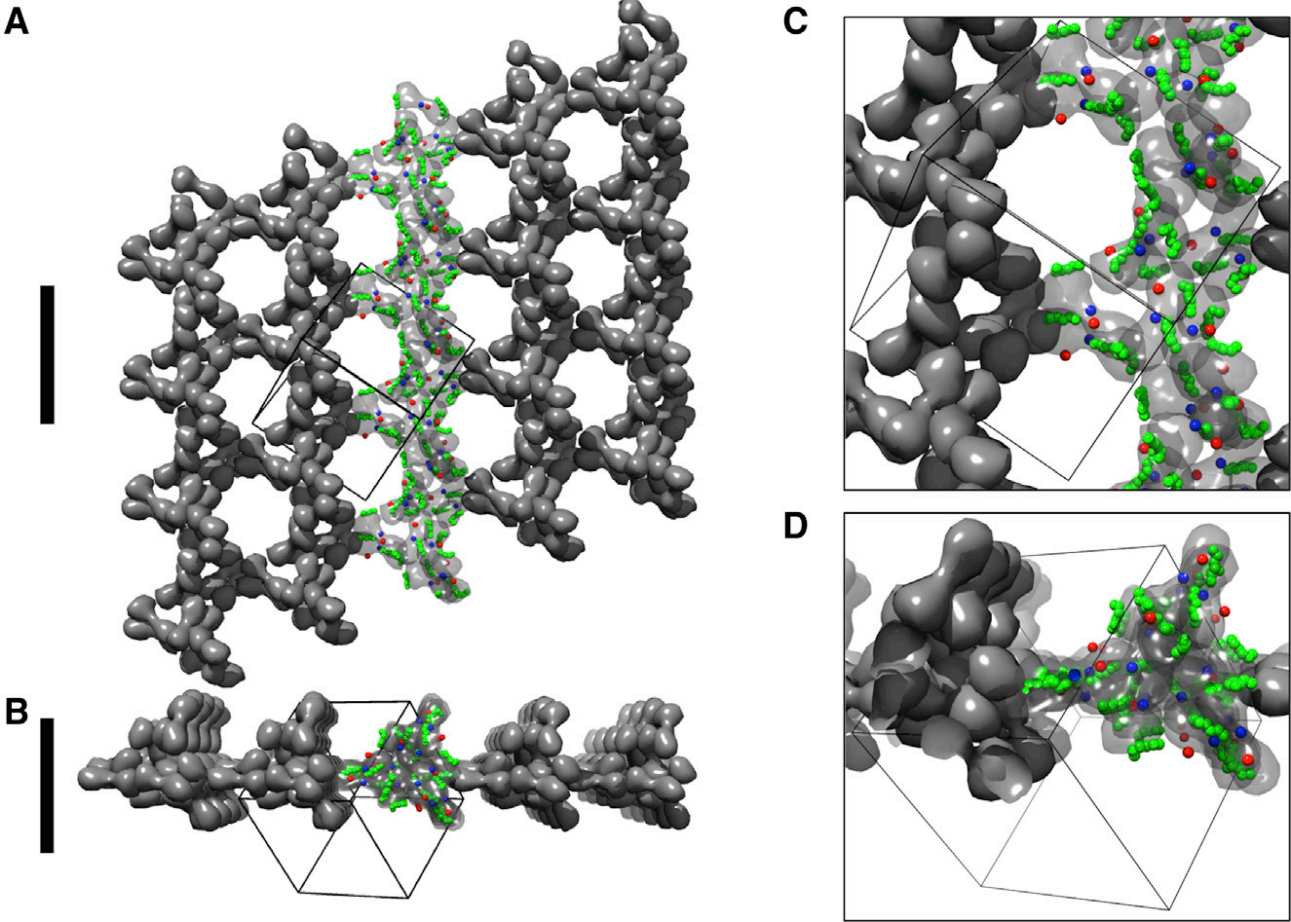
Higher order PDZ1-2 structures. Orthogonal views of a PDZ1-2 net are shown in (*A*) and (*B*), respectively; each PDZ1-2 domain is represented by a surface envelope. (*C*) and (*D*) show an expanded version of (*A*) and (*B*), respectively. An oligomer coincident with the 3_1_ axis of the scaffolding space group is shown as a semitransparent surface within which the RRESEI ligands (C*α* locations shown as *green spheres*), and PDZ1-2 N- and C-termini (C*α* as *blue* and *red spheres*, respectively) are picked out. The interfaces between this highlighted element and parallel oligomers are mediated by PDZ1-PDZ1 contacts. All scale bars, 148 Å, and a unit cell outline for the scaffolding space group is included with each molecular representation. The diagram was rendered with UCSF Chimera. To see this figure in color, go online.

The PSD-95 N-terminal linker (Fig. 1) has been found to be essential for clustering (23). The N-terminal linker is responsible for membrane localization, which is a major factor in maintaining a high concentration of PSD-95 near the membrane. The closely related PSD93 MAGUK has been shown to be palmitoylated at the N-terminus (in rats) (61) and may form complexes similar to those found here. The SAXS experiments indicate that the formation of extended oligomers of PDZ1-2 requires saturating concentrations of type I PDZ peptide ligands. These sequences are found in the cytoplasm at the ends of long unstructured regions at the C-termini of transmembrane ion channels. The ligand-saturating condition is therefore also satisfied close to the surface of a membrane bearing a high density of such channels. Arrangements of PDZ1-2 oligomers that are compatible with a high ligand concentration close to a surface are shown in Fig. 6. Extended oligomers were observed in earlier work with the RRESEI containing the Kir2.1 cytoplasmic domain and full-length PSD-95 (12) with both components in the solution phase (Fig. S12). A PDZ1-2 oligomer coincident with the 3_1_ screw axis of the scaffolding space group (*highlighted* in Fig. 6, *A* and *B*) could form the core element of the Kir2.1-PSD-95 structures. To form a two-dimensional net, PDZ1-PDZ1 interactions are necessary to link within and between these extended oligomers (Fig. 6, *C* and *D*). The ligands required to enhance the interfaces between those copies of PDZ1-2 are also available close to a (membrane) surface (Fig. 6, *B* and *D*).

Scaffolds formed in vivo would therefore be subject to the high concentration of both a ligand sequence and PDZ1-2, which is limited to a volume close to the membrane. This constraint will allow the formation of a net of ligated PDZ1-2, but a deeper three-dimensional array of PDZ1-2 cannot form. GSH is an indigenous cellular peptide, the SAXS work here shows that GSH has similar effects on PDZ1-2-PDZ1-2 association as the RRESEI ligand. These effects are evident at millimolar concentrations of GSH. GSH pervades the cytoplasm of cells at a 0.5–10-mM concentration (62). The GSH ligand is found in the bulk cytoplasm and has the ability to enhance a PDZ1-2-PDZ1-2 dimer interaction, which would support the formation of oligomers at a greater distances from the membrane surface. The SAXS experiments here indicate that GSH-PDZ1-2 oligomers are likely to be more limited in extent. Therefore, structures formed by means of ligated PDZ1-2 scaffolds may be maintained through the integration of GSH but may not be significantly extended by incorporation of GSH.

### The scaffold space group has optimal properties

In common with all cubic space groups, I2_1_3 is isotropic (the same in three orthogonal directions) on the mesoscopic scale. Hence, the packing arrangement dictated by the scaffolding space group could be preserved when following the membrane curvature seen in specialized structures such as synaptic boutons or cylindrical axons. Long bidirectional nets can readily be formed (Fig. 6 *A*) and naturally coincide with a network of crossed 3_1_ screw axes that are parallel to “isotropic” body diagonal directions of the I2_1_3 scaffolding lattice (Fig. 6 *A*). The underlying repeating unit of the order of 14.8 nm determined by the unit cell of the I2_1_3 scaffolding lattice is comparable with separations of PSD-95 seen in tomography (11). A net formed in this way has regularly spaced voids (Fig. 6 *A*), allowing space for other components to integrate with a scaffold.

### Scaffold modulation

**Figure 7:**
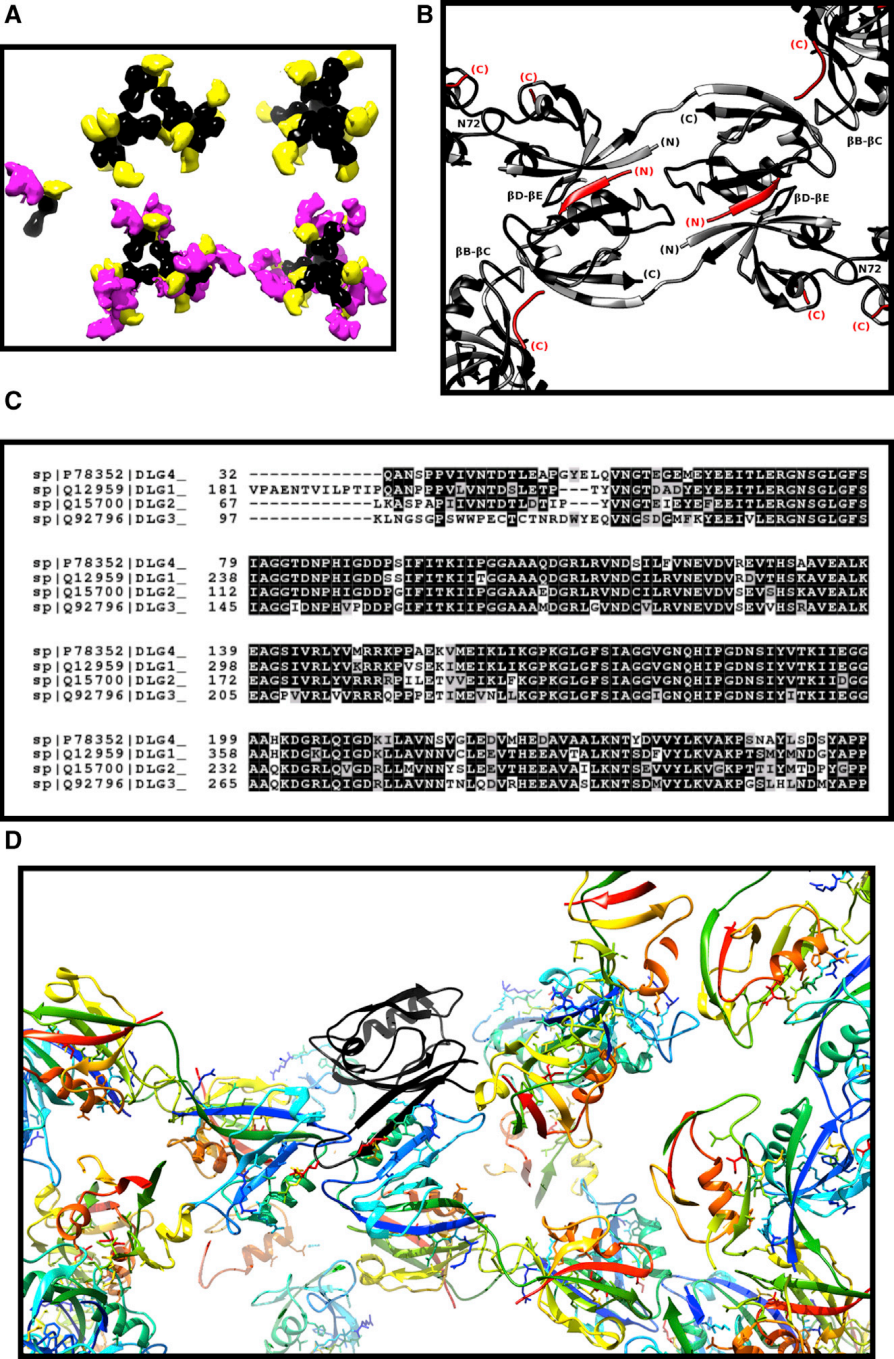
Modulation of the PDZ1-2 scaffold. (*A*) shows views of a dodecamer (Fig. 5*A*, *12e*) of PDZ1-2 drawn from the scaffolding space group decorated with PDZ3 and PDZ3-SH3GK. An isolated PDZ1-2-PDZ3-SH3GK model is shown leftmost. Molecules are represented in a similar way to Fig. 6, with PDZ1-2 in black, PDZ3 in yellow, and SH3GK in magenta. Sequence variation in the PDZ1-2 fragment across the MAGUK proteins (DLG1-4) is represented in (*B*), with the corresponding sequence alignment in (*C*). UniProt codes are shown alongside gene names in the alignment. The PDZ1-2 domains are shown in cartoon form, with the RRESEI ligand in red. The C- and N-termini of each PDZ1-2 are labeled in parentheses, the C-terminus of each RRESEI peptide bound at PDZ1 is labeled, and the N-terminus of each RRESEI peptide bound at PDZ2 is labeled. Selected structural elements at interfaces formed in the packing arrangement are also labeled. The PDZ1:PDZ1 interface is located by residue Asn 72 (N72); the *α*B(PDZ2)-*β*D-*β*E(PDZ1) interface is located by the *β*D-*β*E loop label and the *α*A(PDZ2)-*β*B-*β*C(PDZ1) interface by the *β*B-*β*C loop label. The sequence alignment obtained from the program ClustalW (72) is mapped onto the packing arrangement with the locations of identical residues colored black, similar residues colored gray, and residues with different properties colored white. (*D*) shows a section of the packing arrangement shown in Fig. 6*A*, with PDZ1-2 displayed in a cartoon form in a similar manner to Fig. 1; the nNOS PDZ domain (*black*) is shown added by docking on to the scaffold in association with PDZ1. Docking is accomplished via the hairpin loop of the nNOS PDZ domain inserting into the PDZ1 cleft (shown overlaying a bound RRESEI ligand in the figure). Molecular representations were generated with UCSF Chimera. To see this figure in color, go online.

The PSD-95 protein contains five modules, only two of which are considered in the work reported here; hence, the arrays shown in this work may be affected by the other domains of the protein. The PDZ1-2 module of PSD-95 is bracketed by two long unstructured peptide linkers (Fig. 1), which will allow relative freedom for the PDZ1-2 module to form interactions with other copies of PDZ1-2 close by. However, the closest interaction partner for PDZ1-2, an isolated PSD-95 molecule, will be PDZ3. It is therefore relevant to examine whether the PDZ3 domain could form similar domain-domain interactions to those found in the PDZ1-2 scaffolding space group. The precise affinity of any PDZ-PDZ interaction will be governed by the amino acids forming interfaces, including any associated ligand peptides, but compatible secondary structure elements also need to be in place. PDZ3 has additional secondary structure elements after the *β*F-strand and also has a truncated *β*B-*β*C loop (20). This means that PDZ3 could substitute for PDZ2 in the scaffolding space group (*α*B(PDZ3)-*β*D-*β*E(PDZ1), *α*B(PDZ2)-*β*D-*β*E(PDZ3), and *α*A(PDZ3)-*β*B-*β*C(PDZ1) could all form). PDZ3 can form the equivalent of the PDZ1-PDZ1 interaction (PDZ1-PDZ3) seen in the scaffolding space group; however, the domain could not form an *α*A(PDZ2)-*β*B-*β*C(PDZ3) interaction. This means that PDZ3 cannot fully substitute for PDZ1 in the scaffolding space group. Based upon this simple analysis, PDZ3 is likely to integrate with the scaffold, albeit in a more limited manner compared with PDZ1 and PDZ2 of PDZ1-2. It is important to note that for the oligomers described here (Fig. 5 *A*), all copies of PDZ1-2 form at least two interactions. The avidity due to this multiple interaction means that the dual domain intermolecular interaction is likely to displace any PDZ1-2-PDZ3 intramolecular interaction on the close approach of a second PDZ1-2 unit. As such, the PDZ1-2 domain may form a core scaffold that can be decorated by PDZ3. It is possible to use the properties of the lattice to examine how additional PDZ domains can integrate with the scaffold; Fig. 7 *A* shows the incorporation of the PDZ3 domain from PSD-95 (PDB: 1bef (20)). PDZ3 can enhance the binding between tetramers by forming additional contacts to itself or to copies of PDZ1-2. In the model shown in Fig. 7 *A*, this is the case for 8/12 PDZ3 domains in the modeled dodecamer of PDZ123; the PDZ3-PDZ1-2 contacts could be inter- or intramolecular in nature as restricted by the linker between PDZ1-2 and PDZ3.

A more stringent limitation on the formation of the scaffolding lattice is the incorporation of the PDZ3 and SH3GK domains of PSD-95. Extending the formalism outlined above, it is possible to gain some insight into the formation of whole-MAGUK oligomers. Using the ZO-1 PDZ3-SH3-GK structure (63) as a template, a model of the same region of PSD-95 can be realized by docking the structures of PDZ3 and SH3-GK (64). The modeled PDZ3-SH3-GK structure can then be docked onto the scaffold in the same way as for PDZ3, although, because there is a clear conformational difference between the ZO-1 SH3-GK and PSD-95 SH3-GK, any resulting analysis can only be approximate. This model is shown in Fig. 7 *A* using a PDZ1-PDZ3 contact for docking the PDZ3-SH3-GK addition. It is clear that the full-length protein can be incorporated into extended linear oligomers coincident with a 3_1_ screw axis (Fig. 7 *A*). This type of oligomer is consistent with those observed in earlier electron microscopy images (as illustrated in Fig. S12). The linking of units these together to form a net, shown in Fig. 6, would require alternate conformations of PSD-95 or enzymatic cleavage to remove SH3-GK, PDZ3-SH3-GK, or a combination of the two. MAGUK proteins are substrates for the calpain family of calcium-dependent proteases (65,66), which are implicated in synaptic plasticity (67). Calpain cleavage of PSD-95 occurs in the flexible links between PDZ12 and PDZ3 and between PDZ3 and SH3GK. The catalytic domain of calpain-2 has a type I PDZ binding motif (-FSVL), indicating that the enzyme can bind to type I PDZ domains to enhance the efficiency of target protein cleavage. Thus, modification of PSD-95 by calpain may lead to the formation of two-dimensional nets, shown in Fig. 6.

### Incorporation of other component molecules into a scaffold

An additional feature of the symmetry of the scaffolding arises if one considers breaking the precise symmetry of the PDZ1-2 double dimer (shown in Fig. 5 *A*, *2e*). This asymmetry can be accommodated with a revised P2_1_3 scaffolding space group with an identical unit cell length. Thus, as long as the residues involved in the scaffold contacts are compatible, the packing of heterogenous PDZ1-2 domains can occur in a similar way. Other MAGUK proteins are found alongside PSD-95 in synaptic fractions, including SAP97 (4). The PDZ1-2 regions of the canonical isoforms of PSD-95, SAP97, PSD93, and SAP102 have an amino acid identity of 72%. The PDZ1-2 region of SAP97 has an identity of 89% with PSD-95 but is not localized to the membrane via post-translational modification like PSD-95 (and PSD93). It has been shown that PSD-95 and PSD-93 can heteromultimerize (68); hence, mixed MAGUK oligomers may form via PDZ1-2 interactions. Fig. 7 *B* shows the P2_1_3 packing with the sequence variation across PSD-95, SAP97, PSD-93, and SAP102 (DLG4, DLG1, DLG2, and DLG3, respectively; Fig. 7 *C*) mapped onto the structure. It is evident that the PDZ1-2 scaffold could accommodate heterogeneity in the PDZ1-2 domains. In this way, a cubic scaffolding lattice can also be extended further into the cytoplasm through the incorporation of other MAGUKs. It is interesting to note that the F119 residue implicated in the binding of GSH to the PDZ1 domain of PSD-95 and, in turn, the stabilization of oligomers is not conserved across the MAGUK family. Therefore, although peptide ligand stabilization of heterogeneous PDZ1-2 interactions is possible, GSH integration may not take place for all combinations.

More diverse partner proteins can also associate with a PDZ1-2 scaffold. The association may be either via the incorporation of a compatible C-terminal sequence or through the integration of compatible PDZ domain(s) in the manner discussed (for PDZ3) above. A laminar model of the PSD (6) indicates that the nNOS protein is found in abundance at the cytoplasmic side of a PSD-95-enriched layer. The nNOS protein has an N-terminal PDZ domain that can afford integration with the PSD-95 scaffold, and an interaction has been shown in rats (69). The PDZ domain of nNOS can interact with other PDZ domains via the insertion of a *β*-hairpin into the peptide binding cleft, as resolved in the crystal structure of the domain with syntrophin PDZ (51). Assuming an equivalent mode of interaction takes place with PDZ1-2, the interaction of the nNOS PDZ domain with the PDZ1 domain of PDZ1-2 in the scaffolding space group can be modeled as shown in Fig. 7 *D*. The PDZ1 domain is located at the rim of the voids shown in Fig. 6 *A*; hence, an association can form between nNOS and a core scaffold.

Modulation of the scaffold structure and, in turn, the underlying organization of channels may be also be afforded by relatively minor modifications to the ligand or the PDZ domain though post-translational modification. For instance, the phosphorylation of S and T residues in the type I PDZ binding sequence motifs are likely to affect the formation of oligomers. The Ser residue in in the RRESEI sequence of Kir2.1 has a very high phosphorylation likelihood, as does the equivalent residue at the termini of the NMDA receptor (NetPhos 3.1 sever scores of 0.99 and 0.98, respectively (70)), and although a sequence bound at PDZ2 in the scaffold may be protected from kinases, one bound at PDZ1 is accessible.

## Conclusions

The PDZ1-2 component of PSD-95 is known to be an essential component for receptor clustering at the synapse and presumably other locations, such as juxtaparanodes. This study shows that PDZ1-2 oligomerization can be accomplished by the dual domain and a ligating peptide alone—whole receptor proteins are not required. The PDZ domain itself may be considered to be a modifiable interacting element because the interactions between PDZ domains in PDZ1-2 scaffolds are modulated by the properties of a bound peptide ligand. On binding a suitable peptide ligand, particular structural elements of the PDZ1-2 domain are stabilized with mediation by the GLGF motif, and in turn, the ligated domain favors the formation of oligomers.

The interaction mechanism of the PDZ1-2 domain revealed here is the formation of oligomeric structures that conform to a cubic space group; hence, a systematic rule underscores the highly organized scaffold formed by PDZ1-2. The ubiquitous intracellular peptide GSH can also induce the formation of similar inter-PDZ1-2 oligomers and GSH may also be incorporated into the same scaffold structure.

### Accession numbers

The apo-PDZ1-2- and RRESEI-PDZ1-2-refined structures have been deposited in the Protein Data Bank (PDB; https://www.wwpdb.org) with accession codes PDB: 6spv and PDB: 6spz. The concentrated, diluted, and SEC-SAXS curve data with corresponding oligomer models have been deposited in the Small Angle Scattering Biological Data Bank (SASBDB; https://www.sasbdb.org): for RRESEI-PDZ1-2, accession codes SASBDB: SASDGB5, SASBDB: SASDGC5, and SASBDB: SASDGD; for apo-PDZ1-2, SASBDB: SASDGE5, SASBDB: SASDGF5, and SASBDB: SASDGG5; and for GSH-PDZ1-2, SASBDB: SASDGH5, SASBDB: SASDGJ5, and SASBDB: SASDGK5. A PDB format file representing the asymmetric unit of the PDZ1-2 scaffold can be generated from the chain identifier “A” of any of the oligomer models deposited in the SASBDB. The PDB file header can be generated using space group symmetry I2_1_3 with the unique cell parameter |**a**| = 148 Å.

## Author Contributions

L.B. designed and generated the clones and characterized their expression in *E. coli* bacteria at the Oxford Protein Production Facility and with support from the Oxford Module Consortium. J.C. corrected cloning artifacts via site-directed mutation with advice from Dr. James Birtley (University of Manchester) and carried out pilot purification with the help of Dr. Ronald Burke (Manchester Protein Production Facility). N.A.R. produced and purified the protein samples for each of the studies reported here. Protein crystallization was performed by N.A.R. and C.W.L. X-ray crystallographic data were collected by C.W.L. Crystallographic interpretation was carried out by N.A.R. and S.M.P. ITC was conducted by N.A.R. C.B. and M.P.L.-C. collected SAXS data and carried out initial interpretation. SAXS interpretation was carried out by N.A.R. and S.M.P. The information provided by the 3gls and 3zrt structures of PDZ1-2 was essential to the analysis of SAXS data and the resulting oligomer models presented here. The manuscript was written by S.M.P., with contributions from N.A.R., C.B., and C.W.L.

## Supporting Material

Document S1. Figs. S1–S12 and Tables S1–S2

Document S2. Article plus Supporting Material
