# Supplementary material for: The Dual PDZ Domain from Postsynaptic Density Protein 95 Forms a Scaffold with Peptide Ligand
Source: Biophys J. 2020 Jun 26;119(3):667–89. doi: 10.1016/j.bpj.2020.06.018 (PMC7399497; doi:10.1016/j.bpj.2020.06.018)
Supplement: Document S1. Figs. S1–S12 and Tables S1–S2 [file mmc1.pdf]

Biophysical Journal, Volume *119*

Supplemental Information

The Dual PDZ Domain from Postsynaptic Density Protein 95 Forms a Scaffold with Peptide Ligand

Nazahiyah A. Rodzli, Michael P. Lockhart-Cairns, Colin W. Levy, John Chipperfield, Louise Bird, Clair Baldock, and Stephen M. Prince

Supplementary materials

Table S1. X-ray Crystallography

	apo-PDZ1-2	RRESEI-PDZ1-2
Beamline; detector	Diamond Light Source (DLS) station IO4; PILATUS 2M	DLS IO4-1; PILATUS 6M
Wavelength; crystal-detector distance; image width; exposure; image number	0.920Å; 249.77mm; 0.25^{O}; 0.2s; 720+280	0.979Å; 264.97mm; 0.25^{O}; 0.1s; 400+400
Spacegroup and unit cell	$I4_1$; a=50.38; c=178.27Å	$I4_1$; a=50.50; c=176.37Å
Resolution; Rmerge; I/σ(I). Figures in parenthesis are for the highest resolution shell.	48.48-2.04 (2.09-2.04)Å; 5 (54.7)%; 24.4 (2.8)	48.55-2.08 (2.13-2.08)Å; 7.5 (53.8)%; 14.5 (2.7)
Completeness; multiplicity Figures in parenthesis are for the highest resolution shell.	98.8 (93.8)%; 5.4 (2.8)	99.6 (97.8)%; 3.4 (1.8)
Number reflections (test set); Rcryst; Rfree.	13,259 (735); 22.4%; 26.0%	12,513 (703); 20.0%; 24.0%
Residue range PSD95; Kir2.1 peptide ligand at PDZ1 & PDZ2; GSH; number of water molecules	58-246; n/a & n/a; 1; 40	58-246; 422-427 & 422-427;1; 56
Root mean square deviation bond lengths; bond angles from ideal values.	0.006Å; 0.996^{O}	0.006Å; 1.139^{O}

Table S2. Small angle X-ray Scattering data sets

date	June '15				September '16				Jan '16			Jun '16	
Beamline; geometry; wavelength; q-range	DESY P12 beamline; fixed sample concentration; 1.24Å; ∞ - 0.48Å^{-1}				DLS B21 beamline; fixed sample concentration; 1.0Å; ∞ - 0.4Å^{-1}				DLS B21; Paused Size exclusion run; 1.0Å; ∞ - 0.4Å^{-1}			DLS B21; integrated size exclusion peak; 1.0Å; ∞-0.4Å^{-1}	
PDZ1-2 Data set name	Conc. Apo	Dil. Apo	Conc. +RRESEI	Dil. +RRESEI	Conc. Apo	Dil. Apo	Conc. +GSH	Dil. +GSH	SEC. Apo.	SEC. +RRESEI	SEC.+GSH	SEC. Apo.	SEC.+GSH
Total Protein concentration (mM)	0.72	0.36	0.72	0.36	0.36	0.18	0.36	0.18	<0.18	<0.18	<0.18	<0.18	<0.18
Ligand (Ligand concentration, mM)	N/A	N/A	RRESEI (10)	RRESEI (5)	N/A	N/A	GSH (10)	GSH (16)	N/A	RRESEI (<10)	GSH (<10)	N/A	GSH (5)
Guinier R_g, (error) Å; I(0) arbitrary units	23.76(0.06); 19483	24.81(0.10); 17355	29.7(1.0); 47587	26.6(1.4); 53491	27.0(1.2); 0.12	26.5(2.8) 0.039	28.2(2.2); 0.14	31.1(2.2); 0.072	24.4(0.2); 0.017	23.5(1.3); 0.0096	23.6(0.7); 0.0077	22.9(0.8); 0.012	23.0(1.0); 0.01
P(r) R_g; D_{max} Å	24.6; 92	25.7; 100	31.2; 131	28.6; 125	26.0; 90	25.5; 80	27.6; 95	31.4; 106	24.9; 94	24.0; 87	24.0; 78	23.5; 80	23.4; 77

June'16 data are not included in the detailed analysis – these data gave results equivalent to the corresponding data sets collected in Jan'16.

DESY; Deutsches Elektronen-Synchrotron, Hamburg, Germany. DLS; Diamond Light Source, Didcot, UK.

PDZ1-2 residues 55-246 of human PSD-95 preceded by Gly-Pro (from the cleaved affinity tag); N/A, not applicable; RRESEI, synthetic peptide of corresponding single letter amino acid sequence; GSH, reduced Glutathione; R_g Radius of Gyration; I(0) Intensity at momentum transfer value of 0, P(r) pair distribution function (of distance r); D_{max}, maximum extent of molecule.

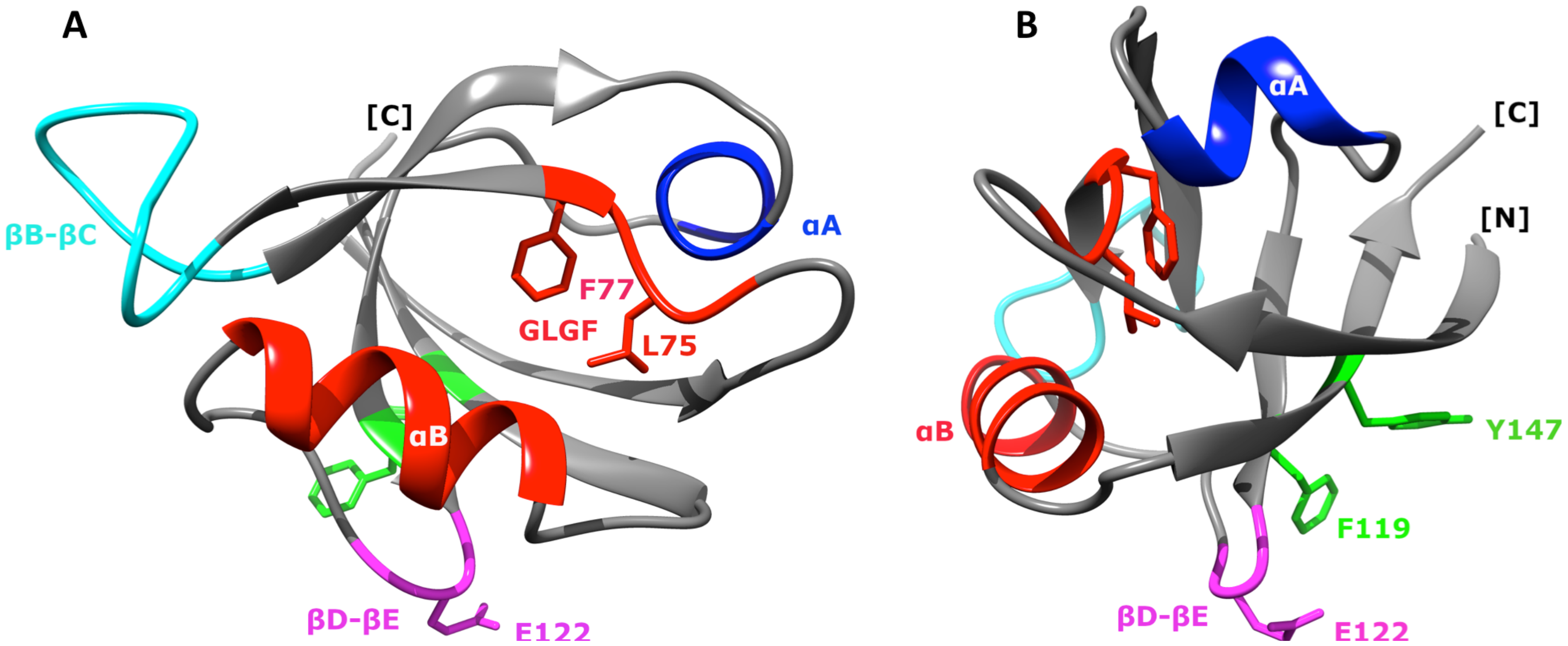

Figure S1. Structural elements of PDZ1 involved in interaction.

A, and B show two orientations of the PDZ1 domain from PSD-95 in grey with structural elements highlighted in colour. The N- and C-terminus of the domain are labelled in square parenthesis. The ligand binding cleft formed between the GLGF motif (labelled, and containing residues Leu 75 and Phe 77) and αB helix is shown in red. The βD-βE loop containing Glu 122 is shown in magenta. The αA helix is shown in blue and the long βB-βC loop in cyan. Residues F119 and Y147 on the βD and βF strands of PDZ1 respectively are shown in green. The PDZ2 domain has a similar structure and also contains a GLGF motif, αA helix αB helix, and a long βB-βC loop.

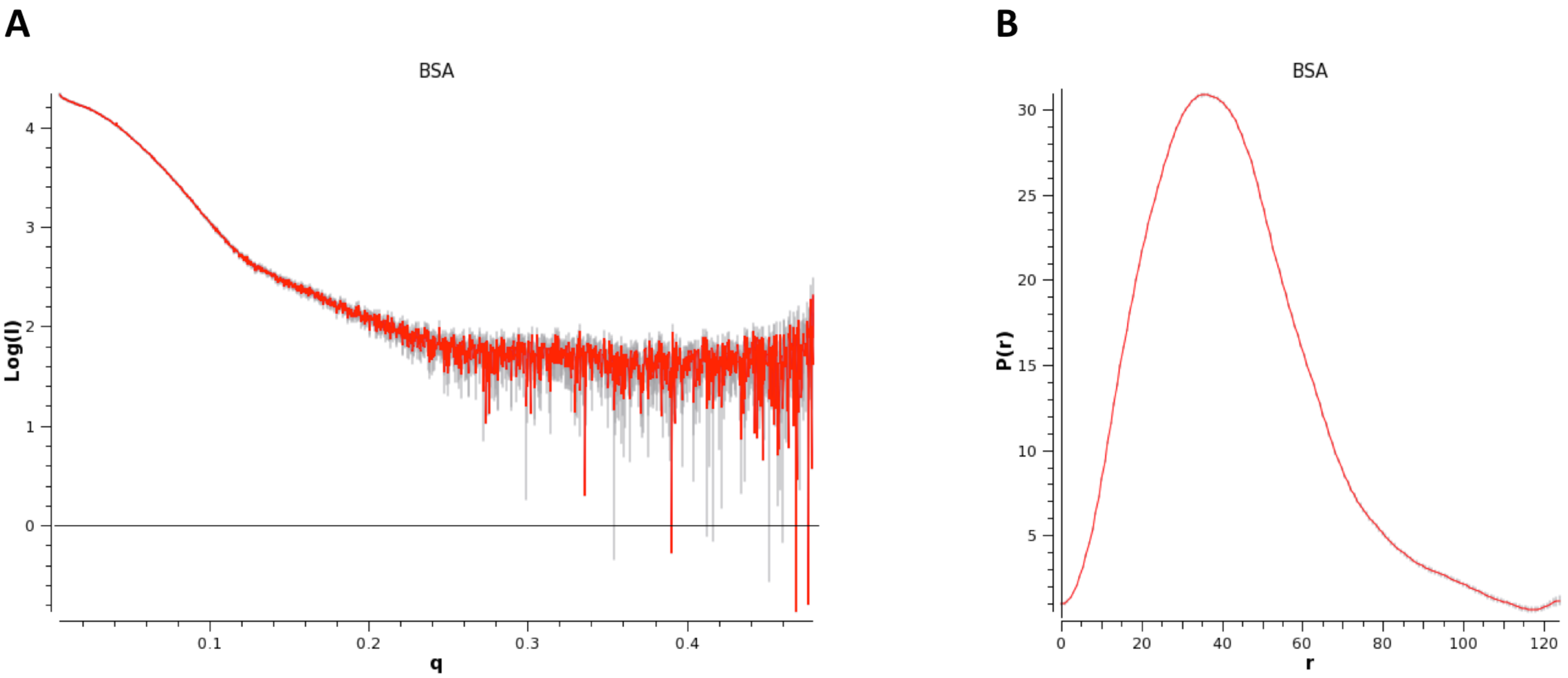

Figure S2. Bovine Serum Albumen (BSA) SAXS data collected at DESY P12, June'15.
Scattering data from a sample of Bovine Serum Albumin at a concentration of 5.2 mg ml^{-1} in HEPES buffer was measured alongside RRESEI-PDZ1-2 and apo-PDZ1-2 data sets at beamline P12 of the Petra PIII (June 15 data). Background subtracted scattering data plotted as Log(I) versus momentum transfer q ($Å^{-1}$) are shown in A. The corresponding pair distance distribution curve is shown in B. P(r) Data points are shown in red, error bars in black. P(r) is plotted against distance, r (Å units) ($P(0)=P(D_{max})=0$ is not imposed).

Values of I(0) and R_g for BSA were obtained from the Guinier plot as $1.879{\times}10^4$ and 33.1 Å. Using the standards obtained for BSA and the I(0) values given in Table S2 the derived concentrations for apo-PDZ1-2 were 5.4 and 4.8 mg ml^{-1} for Jun'15 concentrated and diluted samples respectively. For RRESEI-PDZ1-2 the I(0) derived concentrations were 13.2 and 14.8 mg ml^{-1} for concentrated and diluted samples respectively. These figures are clearly at variance with the dilutions – in particular for the RRESEI-PDZ1-2 data where the derived concentration is higher for the diluted sample. The conversion of the individual curves to an absolute scale would propagate these anomalies.

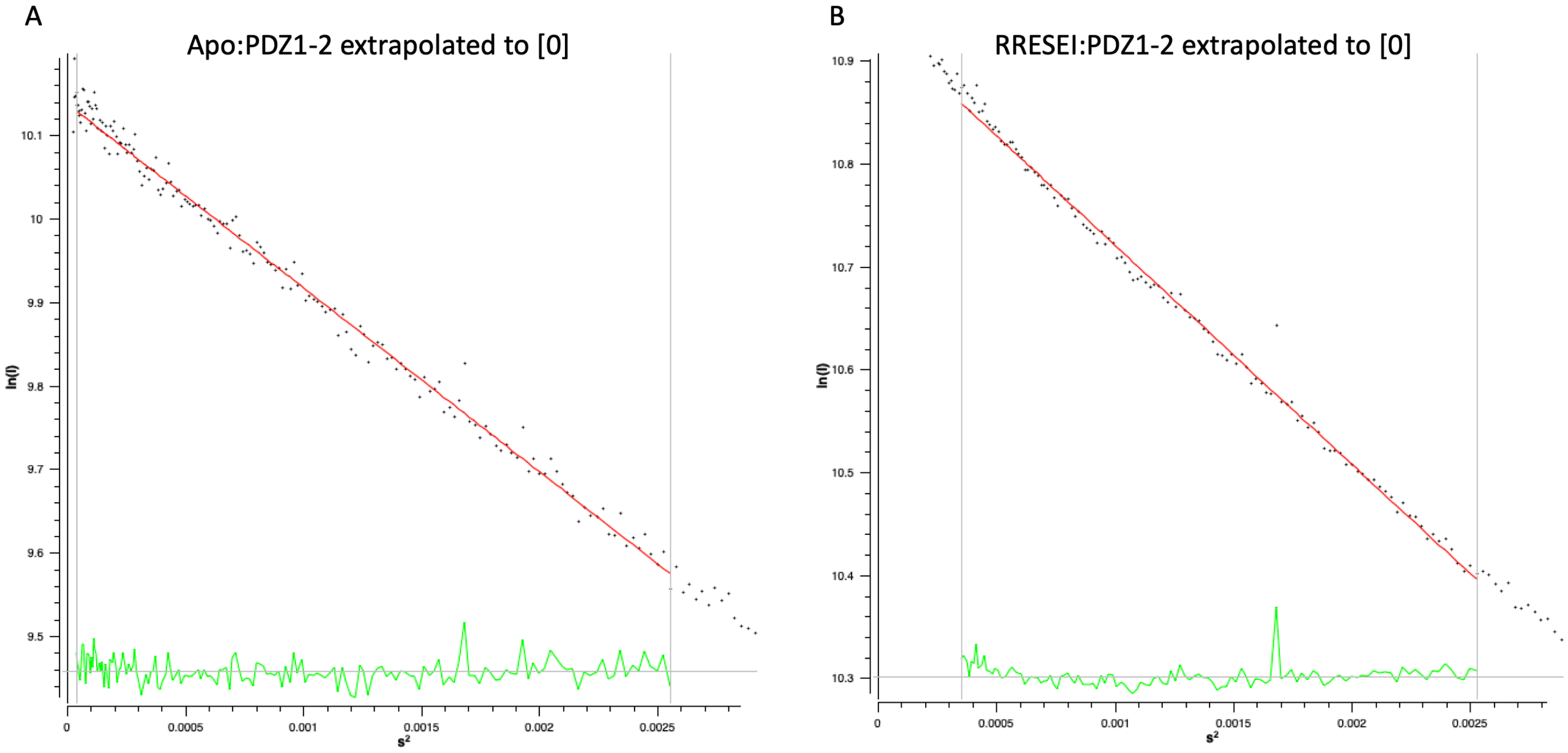

Figure S3. Guinier plots for apo-PDZ1-2 and RRESEI-PDZ1-2 data extrapolated to zero concentration.

The Guinier plots for Apo-PDZ1-2 and RRESEI-PDZ1-2 data extrapolated to zero concentration are shown in A and B respectively. Data points are black and the fitted line is shown in red, s^2 denotes the square of the momentum transfer. The Green curve plots the difference between the fit and observed data. The I(0), R_g are 25281(42), 25.71(20)Å and 56054(113), 25.28(36)Å for Apo-PDZ1-2 and RRESEI-PDZ1-2 respectively.

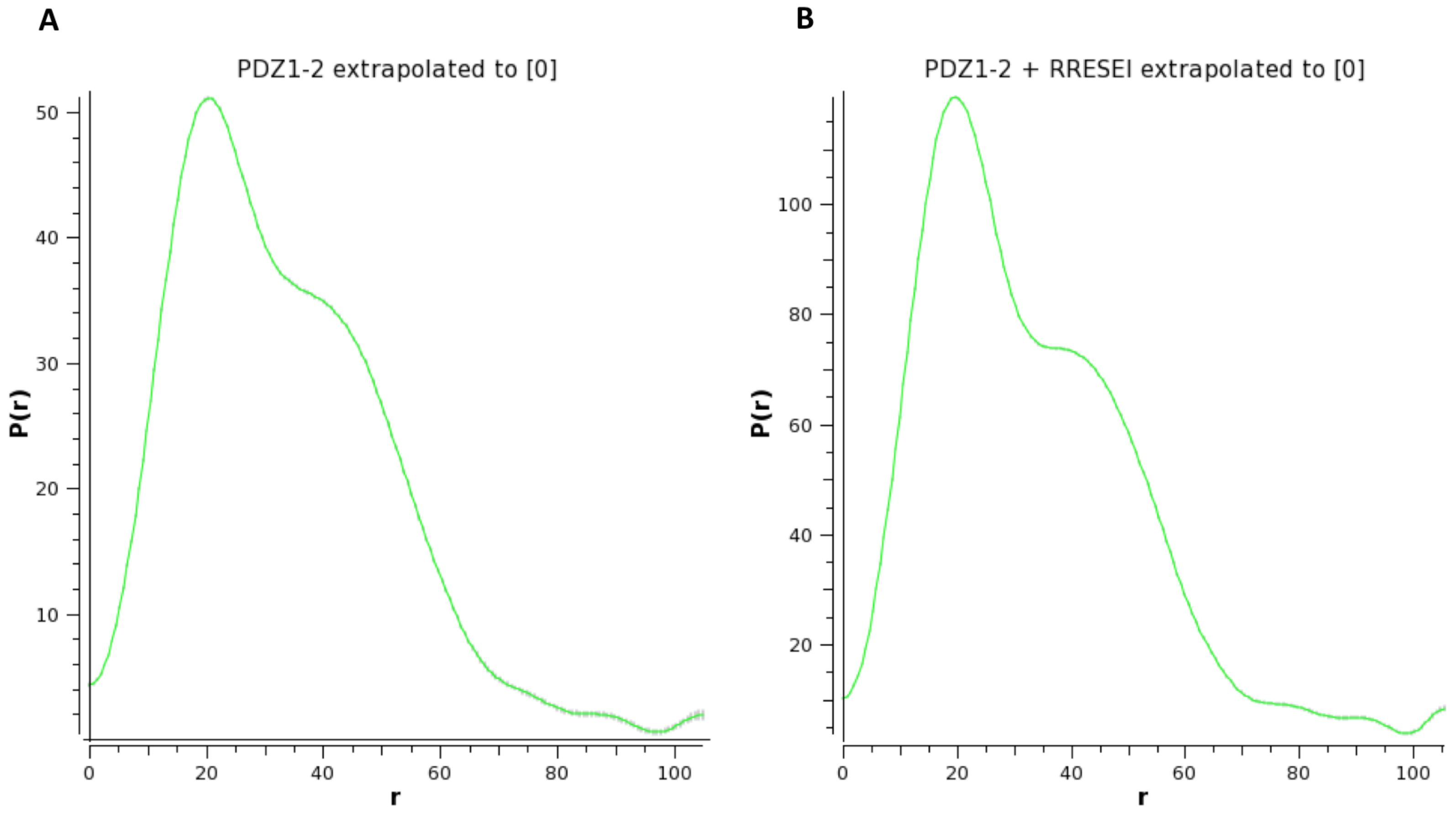

Figure S4. Pair distance distribution curves for apo-PDZ1-2 and RRESEI-PDZ1-2 extrapolated to zero concentration.
Pair distance distribution P(r) curves for apo-PDZ1-2 (A) and RRESEI-PDZ1-2 (B) data extrapolated to zero concentration plotted against distance, r (Å units). Data points are shown in green, error bars in black. $P(0)=P(D_{max})=0$ was not imposed in the calculation of P(r).

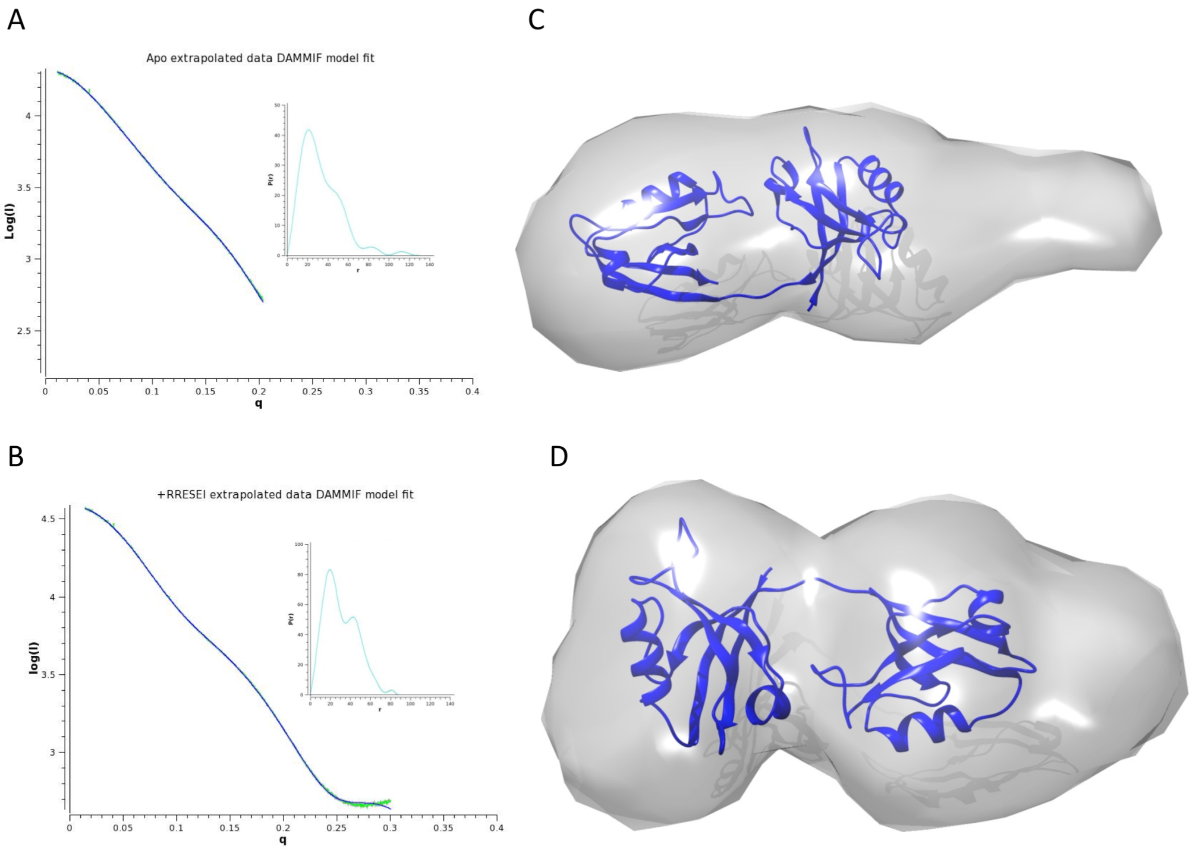

Figure S5. *Ab-initio* modelling from apo-PDZ1-2 and RRESEI-PDZ1-2 data extrapolated to zero concentration.
The fit of a representative dummy atom model derived from the program DAMMIF to the extrapolated data is shown in A for apo-PDZ1-2 and B, RRESEI-PDZ1-2. The curve calculated from the dummy atom model is shown in blue, data points in green, q in units of Å^{-1}. Inset are the corresponding pair distance distribution curves P(r) plotted against distance in cyan, r (Å units) (here $P(0)=P(D_{max})=0$ was imposed). The corresponding dummy atom models are shown in C (apo-PDZ1-2) and D (RRESEI-PDZ1-2). DAMMIF models are represented as an envelope with the PDZ1-2 crystal structure reported here docked for scale. The envelope resolution matches the high q limit of the (truncated) data used. Model diagrams produced in UCSF Chimera.

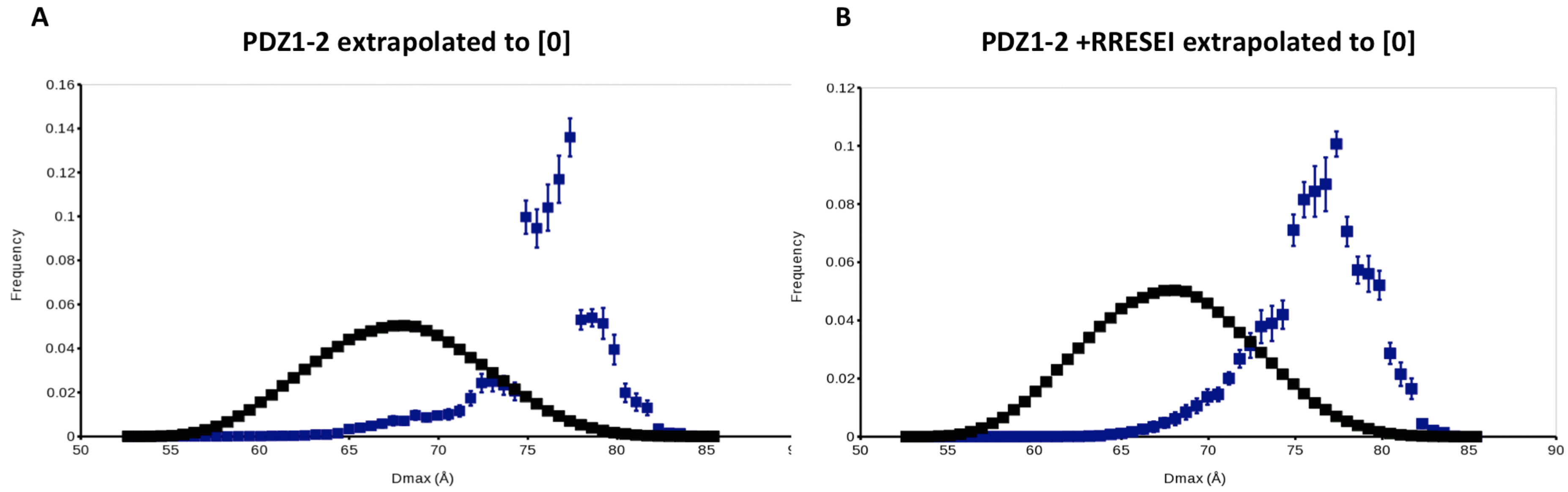

Figure S6. D_{max} Ensemble model analysis histograms.

The D_{max} histograms for the models selected in ensemble model analysis are shown for SAXS data extrapolated to infinite dilution (A, apo-PDZ1-2 and B, RRESEI-PDZ1-2). These graphs correspond to those shown for R_g in Figure 2 E, F. The model population is plotted on the ordinate and D_{max} on the abscissa, black points are pool models and blue points are models selected from the pool, with error bars calculated as the standard deviation of 20 duplicate runs.

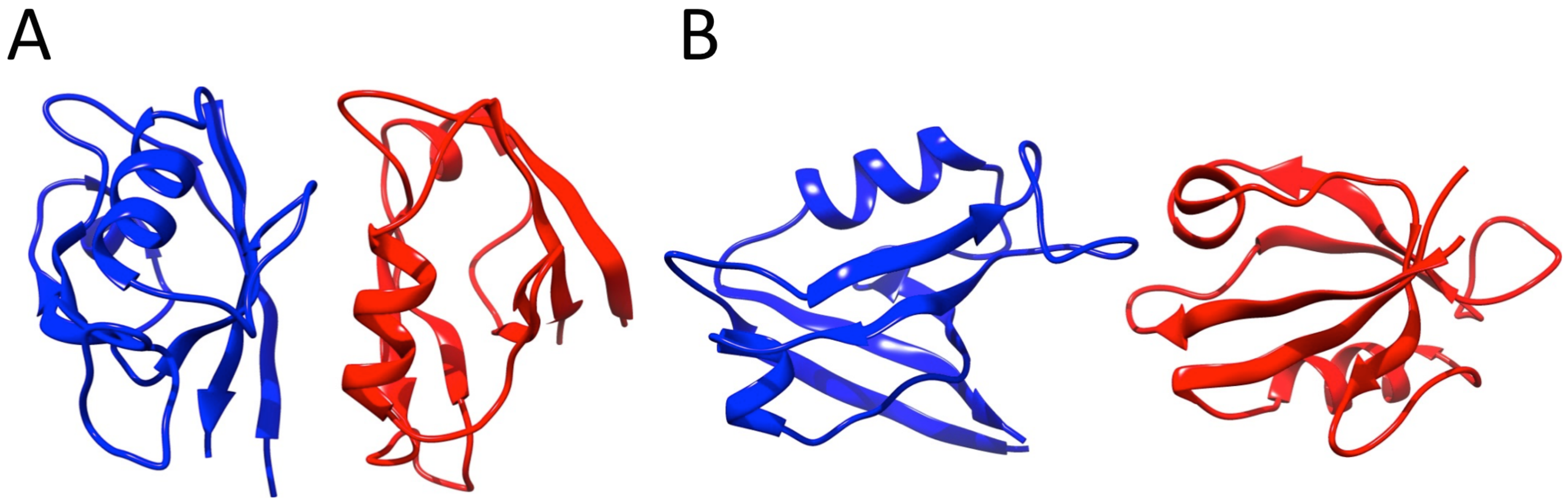

Figure S7. Crystal contacts used in the construction of oligomeric models.
The structures of PDZ domains are shown in cartoon form with secondary structure elements highlighted in a similar manner to Figure 1. PDZ1 is coloured blue and PDZ2 red. The αB(PDZ2)-βD-βE(PDZ1) interface present in the 3zrt and 3gsl crystal structures is shown in A. The αA(PDZ2)-βB-βC(PDZ1) interface from the 3gsl crystal structure is shown in B.

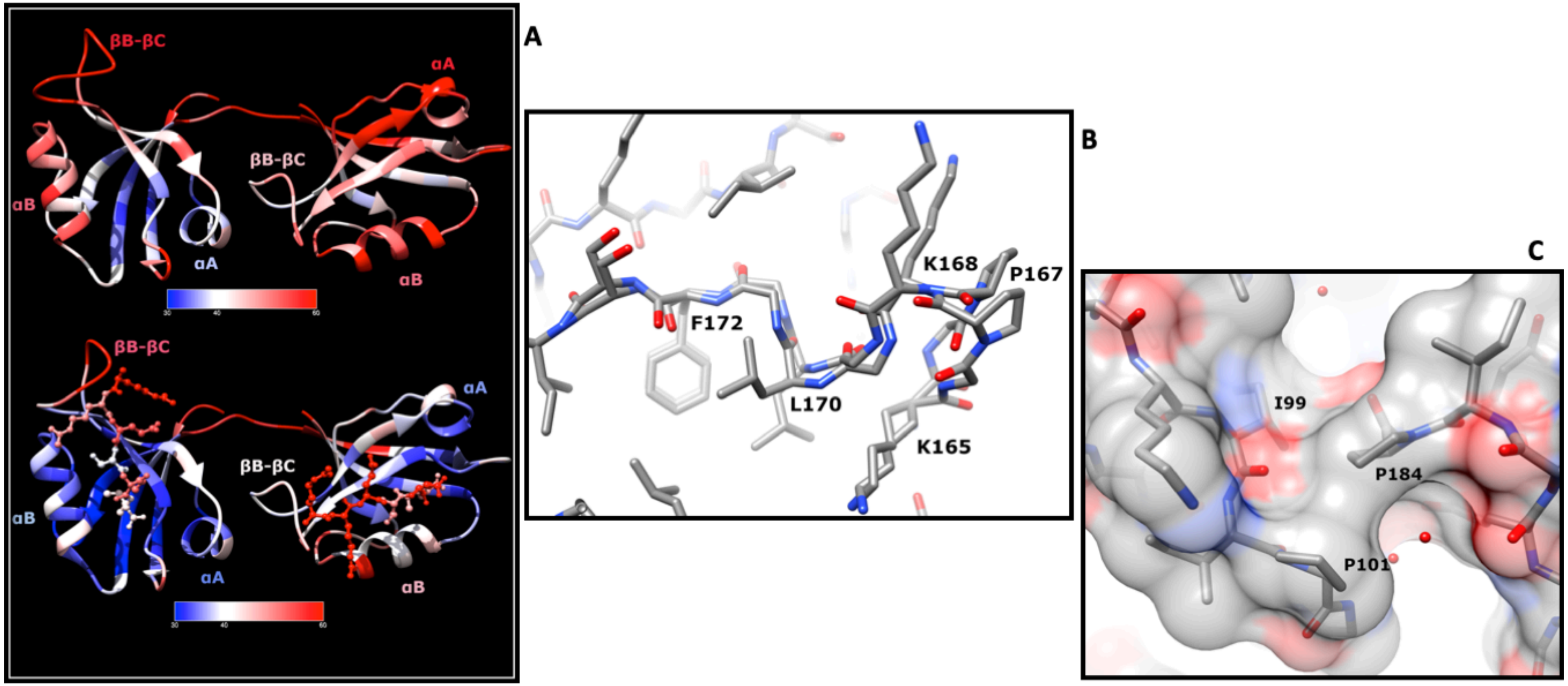

Figure S8. The relationship between contacts and order in the PDZ1-2 crystal structure.
For panel A the structures of apo-PDZ1-2 (top) and RRESEI-PDZ1-2 (bottom) were re-refined without alternate conformations and with isotropic B-factors- The mean isotropic B-factors of residues are represented on the colour scale indicated. PDZ1-2 is shown in cartoon form as in Figure 1, RRESEI is in ball-and stick form, GSH is omitted. The αA and αB helix, and βB-βC loop of each domain is labelled. In B the refined dual conformation of the GLG(F) containing βA-βB loop of apo-PDZ2 is shown the structure is shown in stick form with colours representing the connected atoms (C, grey; N, blue; O, red). In C the PDZ1(αA)-PDZ2(βB-βC) contact formed in both apo-PDZ1-2 and RRESEI-PDZ1-2 structures is shown with a molecular surface overlaid to indicate the exclusion of water from this contact. The stick representation of PDZ1-2, the overlaid semi-transparent surface and water oxygens (red spheres) are coloured according to atomic species as in B.

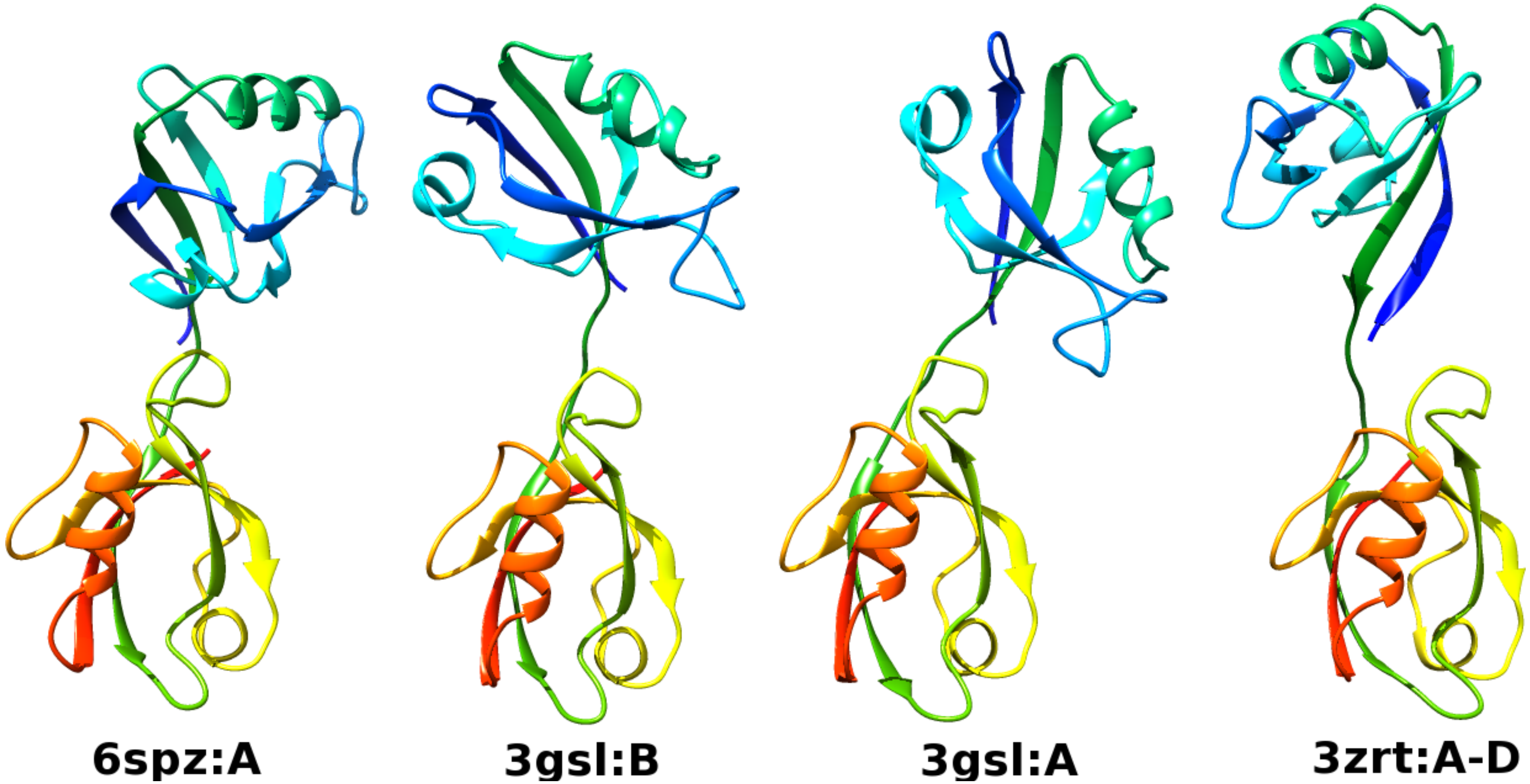

Figure S9. Tandem PDZ1-2 domain crystal structures.

The four crystal structures of PDZ1-2 are shown represented in cartoon form in a similar way to Figure 1 with labels of the form "PDBID:chainID". The compact form of PDZ1-2 from human PSD-95 reported here is shown labelled as 6spz:A; the two conformations of PDZ1-2 from rat PSD-95 3gsl:A and 3gsl:B, and the extended version of human PSD-95 3zrt:A-D.

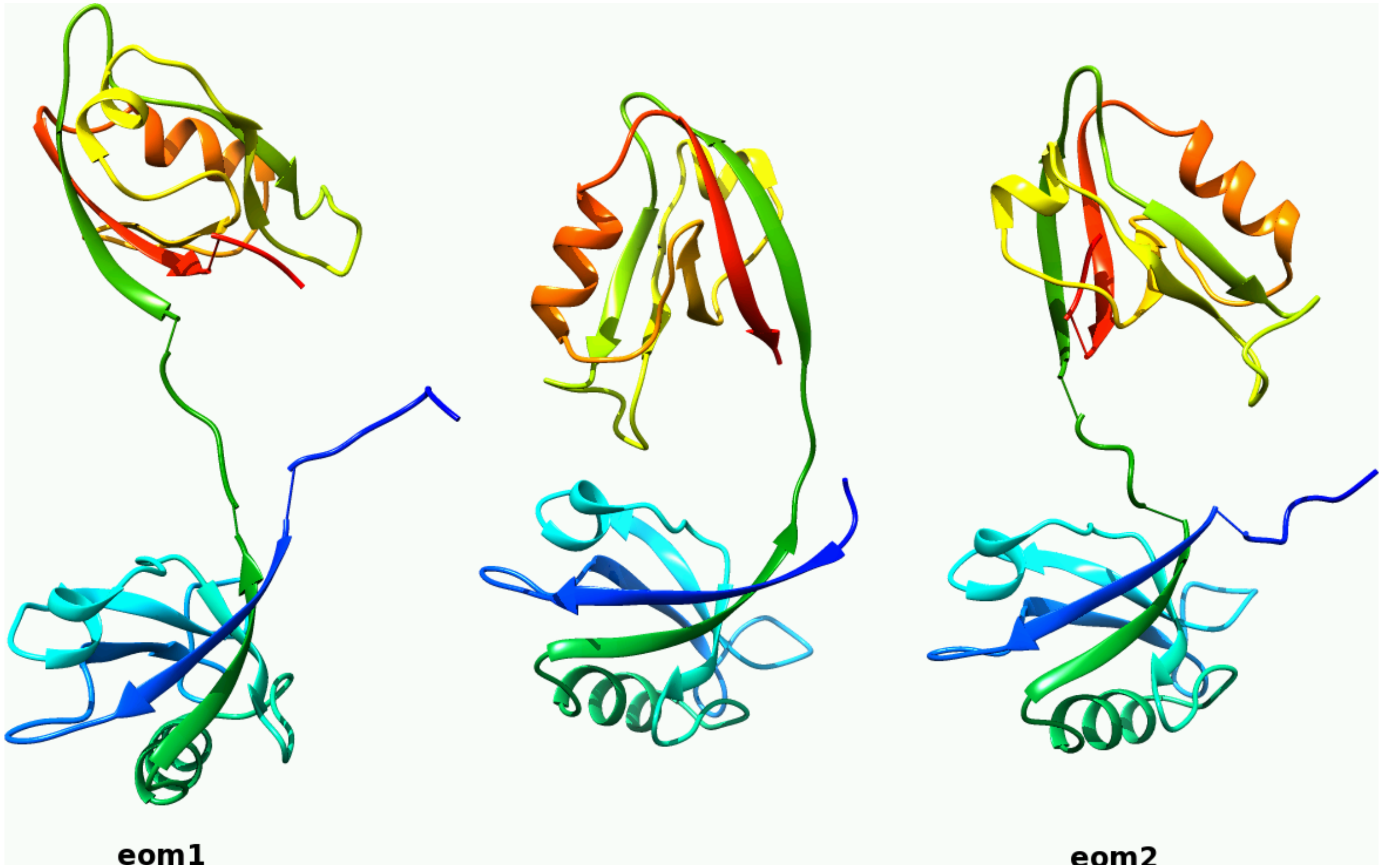

Figure S10. The eom1 and eom2 models used in oligomer-based fitting.

The compact form of PDZ1-2 (corresponding to the crystal structure reported here) is shown in the centre. Molecules are represented in a similar fashion to Figure 1. C-terminal, N-terminal and PDZ1-PDZ2 connecting Cα loop models added in the EOM analysis for eom1 & 2 are shown connected by thin bonds.

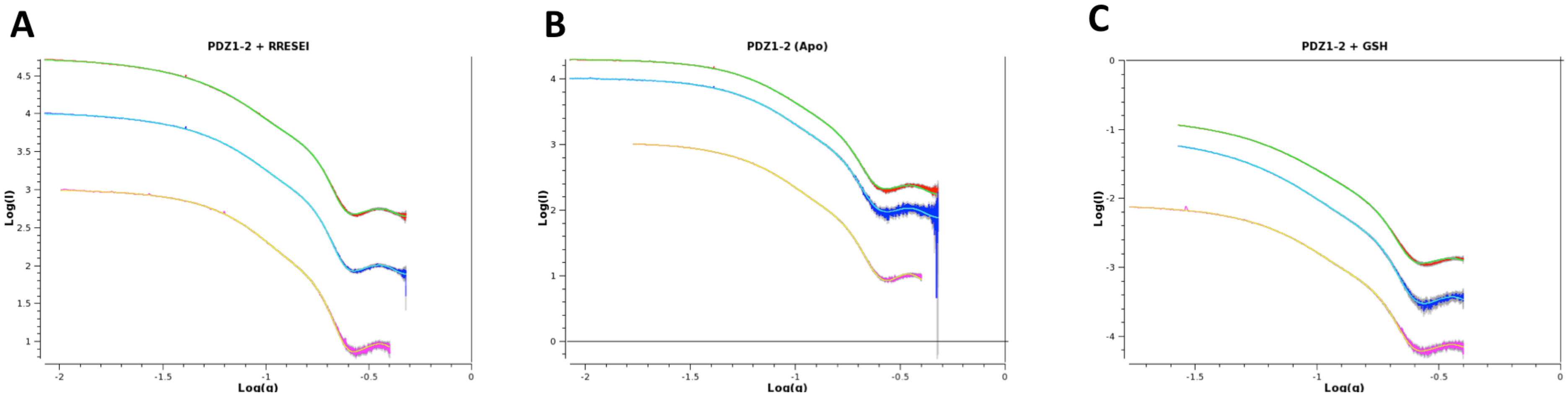

Figure S11. Log(I) versus Log(q) plots of the final oligomer fits.

Transformed plots of RRESEI-PDZ1-2, A; apo-PDZ1-2, B, and GSH-PDZ1-2, C, corresponding to those in Figure 5 are shown. High concentration data curves are shown in red with fitted curves in green, dilutions in blue with fitted curve in cyan and SEC-fractionated samples in magenta with fitted curves in yellow. A multiplication factor has been applied to the raw data in some cases to separate curves along the ordinate (Log(I)) axis.

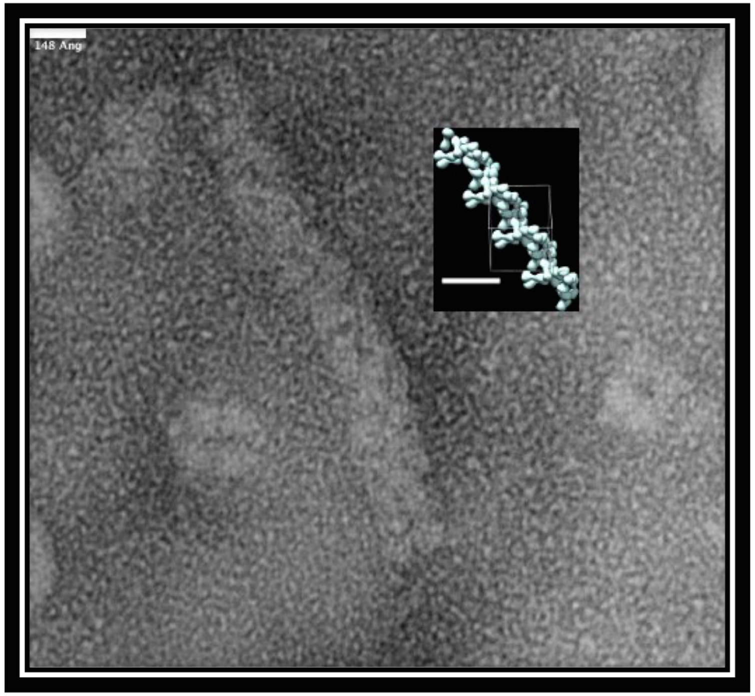

Figure S12. Higher order complexes formed between PSD-95 and the cytoplasmic domain of Kir2.1.

The electron micrograph shown was selected from data for PSD-95 and Kir2.1 complexes (1) and rendered with ImageJ software (2), large complexes are presumed to be formed from full-length PSD-95 and the tetrameric cytoplasmic domain of Kir2.1 (all components in the solution phase, expressed in *E. coli* and assembled *in vitro*). The inset is an oligomer of PDZ1-2 drawn from the scaffolding Spacegroup and coincident with the 3_1 screw axis shown as a molecular surface in a similar manner to Figure 6. A scalebar for the micrograph is shown top left (148 Å) with a matching scalebar on the molecular image – the inset is on approximately the same scale as the micrograph.

Supporting References

1. Fomina S, Howard TD, Sleator OK, Golovanova M, O'Ryan L, Leyland ML, et al. Self-directed assembly and clustering of the cytoplasmic domains of inwardly rectifying Kir2.1 potassium channels on association with PSD-95. Biochimica et biophysica acta. 2011;1808(10):2374-89.
2. Schneider CA, Rasband WS, Eliceiri KW. NIH Image to ImageJ: 25 years of image analysis. Nature methods. 2012;9(7):671-5.
